# Redefining multi-target weather forecasting with a novel deep learning model: Hierarchical temporal convolutional long short-term memory with attention (HTC-LSTM-Attn) in Bangladesh

**DOI:** 10.1371/journal.pone.0342431

**Published:** 2026-03-23

**Authors:** Md Anamul Kabir, Chatak Chakma

**Affiliations:** 1 Department of Electronics & Communication Engineering, Khulna University, Khulna, Bangladesh; 2 Dhaka Power Distribution Company Ltd (DPDC), Dhaka, Bangladesh; Duy Tan University: Dai Hoc Duy Tan, VIET NAM

## Abstract

Bangladesh, being a country that relies on agriculture, has its agricultural sector contributing around 20% to the Gross Domestic Product (GDP) and providing employment opportunities for nearly 60% of the population. The study proposes a novel multi-step forecasting deep-learning framework designed to forecast the maximum temperature and humidity of 24 cities, called Hierarchical Temporal Convolutional Long Short-Term Memory with attention (HTC-LSTM-Attn). The model contains hierarchical temporal convolutions (HTC) for extracting multi-scale patterns, bidirectional LSTMs for sequential dependencies, and an attention mechanism for learning to weigh time steps differently, while parameters are optimized with Keras tuner. Using data from Bangladesh Agricultural Research Council (BARC) from 1961 to 2022 and rigorous preprocessing with seasonal features and lagged statistics, the model obtains temperature results for with a Mean Absolute Error (MAE) of 0.8178 °C, Root Mean Squared Error (RMSE) of 0.9718 °C, R-squared (R²) of 0.8527, and Mean Absolute Percentage Error (MAPE) of 2.8823% and humidity with MAE of 2.4693%, RMSE of 3.2442%, R² of 0.7228, and MAPE of 3.1757% values on a strict temporal test set (19 stations, 2016–2022). On a spatial hold-out test set (5 unseen stations, 2016–2022) performance remains robust, temperature with MAE of 0.9587 °C, RMSE of 1.1898 °C, R² of 0.8342, and MAPE of 3.1534% and humidity with MAE of 2.4796%, RMSE of 3.2119%, R² of 0.6561, and MAPE of 3.2045% consistently surpassing performance measures of models Gated Recurrent Unit (GRU), Long Short-Term Memory (LSTM), Convolutional Neural Network (CNN), Decision Tree Regression (DTR), Convolutional Neural Network – Long Short-Term Memory (CNN-LSTM) and latest transformer-based architectures (Autoformer, FEDformer, TimesNet, and Pyraformer). These results have shown robust spatial performance with the respective improvements needed in agricultural planning and disaster management in Bangladesh, in its vulnerable climate context.

## 1 Introduction

Weather forecasting is a important concern in Bangladesh, an agricultural country that counts agriculture as contributing about 20% to the GDP and 60% of the population’s livelihood [1,2]. It is also essential for agriculture, disaster management, and public health [3–5]. Agronomic activities in the country are highly sensitive to weather variability, including rainfall, temperature, humidity, and wind speed, which have direct effects on crop production, irrigation management, and food security. For instance, heat stress alone in Dhaka results in losses of labor productivity worth $6 billion per annum, which is more than 8% of the annual labor production of the city [6]. In addition, Bangladesh’s tropical monsoon climate greatly aggravates these problems; the monsoon season regularly brings in floods, ruining crops and infrastructure [4]. Recent studies have advanced flood mapping and return period estimation in the country, underscoring both the severity and increasing predictability of flood hazards—e.g., Giezendanner et al. [7], who synthesized two decades of flood mapping efforts, and Saunders et al. [8], who evaluated multiple environmental data streams to refine flood risk estimates. Moreover, with temperatures increasing due to climate change, heatwaves have become more pronounced, particularly in the coastal zones of Chittagong and Cox’s Bazar [9].

With extreme weather events like floods and cyclones causing economic losses of about $1–2 billion every year [9], effective forecasting is needed to enable timely disaster preparedness. For example, the 2020 floods affected over 5 million people causing widespread devastation of rice crops, underscoring this need. The World Bank [10] notes that climate change is increasing the frequency and intensity of these events, thereby requiring the use of sophisticated forecasting techniques for disaster risk mitigation and sustainable development. Conventional means of weather forecasting, on the other hand, such as numerical weather prediction (NWP), have increasingly not been assisting with the complex and fluid weather patterns of Bangladesh [6,11]. These models based on physics fail to adequately describe the non-linear relationship that arises in weather data, especially in a region endowed with monsoonal, riverine, and coastal influence [12].

Over the past couple of decades, the landscape of forecasting witnessed the emergence of ML and DL as potent alternatives capable of modeling complex patterns and improving forecast accuracy. Studies have applied ensemble ML models like Decision Tree Regressor (DTR) and Category Boosting (CatBoost) [1] and deep learning models like Long Short-Term Memory (LSTM), Gated Recurrent Unit (GRU), and Convolutional Neural Networks (CNN) [6,13] in forecasting weather parameters for Bangladesh. Nonetheless, they did not account for finer temporal patterns and aspects that need to be in focus for any accurate forecasting of any dynamic weather events in the area.

In this research paper, we introduce a new framework which is based on deep learning Hierarchical Temporal Convolutional Long Short-Term Memory with Attention (HTC- LSTM-Attn) to remedy the limitations of the earlier models. The proposed HTC-LSTM- Attn model employs a mixture of hierarchical temporal convolutions (HTC) to extract multiscale temporal features, bidirectional LSTMs to model sequential dependencies with an attention mechanism to give better importance to the most informative time steps. Validate it between 1961 and 2022 is a weather dataset, which recorded the collection from the Bangladesh Agricultural Research Council (BARC) [14], used in simulating the conditions in maximum temperature and humidity forecast across 24 cities such as Bangladesh: Dhaka, Chittagong, Sylhet, and others. Model tuning was conducted using Keras Tuner to obtain optimal hyperparameter settings, including 96, 64, and 96 filters for HTC layers, followed by a total of 96 LSTM units and a dense layer with 256 units. This study shows that the HTC-LSTM-Attn model could produce the following performance values: for temporal tests we got, Mean Absolute Error (MAE) of 0.8178 °C, Root Mean Squared Error (RMSE) of 0.9718 °C, R-squared (R²) of 0.8527, and Mean Absolute Percentage Error (MAPE) of 2.8823% for temperature and humidity with MAE of 2.4693%, RMSE of 3.2442%, R² of 0.7228, and MAPE of 3.1757%; additionally, on a spatial hold-out test set temperature with MAE of 0.9587 °C, RMSE of 1.1898 °C, R² of 0.8342, and MAPE of 3.1534% and humidity with MAE of 2.4796%, RMSE of 3.2119%, R² of 0.6561, and MAPE of 3.2045%. The performance is higher than existing GRU, LSTM, CNN, Conv1D, DTR, CNN-LSTM and latest transformer-based architectures (Autoformer, FEDformer, TimesNet, and Pyraformer) models. Visual comparison of temperature and humidity is given at a number of stations which further builds this claim. In order to maintain scientific rigor and prevent data leakage, we follow a strict spatio-temporal evaluation protocol: using data from 1961 to 2012 for training (19 stations), data from 2013 to 2015 for validation (the same 19 stations), and data from 2016 to 2022 for testing across: (i) the same 19 stations (temporal generalization), and (ii) 5 held-out stations (spatial generalization). This configuration—supported by the recent benchmarks such as WEATHER-5K [15]- ensures that there is no overlap, whether in time or space, between the training and test phases. More detailed metrics are found at the station level in S1 Appendix, and they indeed convince further of the performance consistency achieved across all regions.

This research has the following contributions:

1. A newly designed HTC-LSTM-Attn model specifically for multi-target weather forecasting in Bangladesh by using Hierarchical Temporal Convolutions, Bidirectional LSTMs, and an attention mechanism along with the detailed mathematical formulations provided.
2. Very detailed pre-processing of 1961–2022 weather datasets, including exclusion of flat stations, extraction of seasonal components, creation of lagged-rolling statistics, to add more value to model performance.
3. Hyperparameter tuning through Keras Tuner to optimize architecture for robustness and accuracy.
4. Comparative evaluations done against state-of-the-art models (i.e., LSTM, GRU, Conv1D, DTR, CatBoost, CNN-LSTM, Autoformer, FEDformer, TimesNet, and Pyraformer) assuring sound superiority of the HTC-LSTM-Attn model through numerical metrics as MAE, RMSE, R², MAPE and visual comparisons across multiple stations against which half-station-wise results can be seen in the appendix.

The remaining of the paper is structured as follows. Section 2 discusses related work; Section 3 presents the description of the data; Section 4 gives details of the method with equations; Section 5 presents results and discussions and includes visual comparisons between methods; Section 6 concludes the paper and gives future research directions; and the S1 Appendix presents metrics of evaluation on the station level and a preprocessing detail explanation in S2 Appendix.

## 2 Related works

Recently, the use of machine learning and deep learning techniques in weather forecasting has drawn enormous attention even in Bangladesh. The reason for this interest is that the pattern of weather in Bangladesh becomes more complicated and dynamic with the coming of monsoons, river systems, and coastal effects. Mahabub et al. [1] describes for efficient ensemble ML algorithms for forecasting for rain in Bangladesh based on a data sample collected from the Bangladesh Meteorological Division (BMD) between 2012 and 2018. This study compares several regression algorithms, such as Support Vector Regression (SVR), Linear Regression, Bayesian Ridge, Gradient Boosting (GB), Extreme Gradient Boosting (XGBoost), Category Boosting (CatBoost), Adaptive Boosting (AdaBoost), K-Nearest Neighbours (KNN), and Decision Tree Regressor (DTR). Results revealed that DTR and CatBoost outperformed their peers. Compared to an MAE of 5.17 mm for rainfall forecasting in 2018 achieved by DTR, CatBoost had an MAE of 0.426 °C relative to high temperature for data from 2012 to 2017. However, the study cannot use the traditional machine learning methods on the dataset to represent the temporal dependencies of weather data, making it an ineffective approach in capturing the dependencies. Moreover, they used relatively small dataset for their research.

Mumu et al. [6] set out to delve into deep learning methods for multi-target weather predictions in Bangladesh focused on maximum temperature, rainfall, and humidity in eight different cities, namely Dhaka, Mymensingh, Rajshahi, Rangpur, Khulna, Barisal, Sylhet, and Chittagong. The specific dataset extended from 1961–2022 and was collected from the BARC Climate Information Management System. Three deep learning models were then put to test, including LSTM, GRU, and Conv1D. In the end, GRU gave the overall best results, and demonstrated strong performance for rainfall with an MAE of 74.42 mm. The LSTM on its own performed strongly in the maximum temperature prediction with an MAE of 1.68 °C and the Conv1D scored best for humidity prediction with an MAE of 3.47%. In spite of these notable strides forward, their frameworks did not implement multi-scale temporal patterns and consideration of critical time steps which increases predictions in a region that covers diverse weather patterns like Bangladesh.

Mahabub et al. [16] used different models for weather forecasting in Bangladesh based on a BMD dataset of 2012–2017. Among those DTR achieved best results with the value of 0 for MAE,MSE and MAPE. But achieving 0 is not feasible. On the other hand, KNN was reaching an MAE score of 0.78 °C for temperature, MSE score is 1.11°C with a relatively good MAPE 2.54% in comparison with our model and MSE of 6.37 for humidity by linear regression achieved by their work. It lacked the time series dependency with essential attention mechanism that can weigh relevant time steps for forecasting. Moreover, they used relatively very small dataset. Due to the results from their work, it showed some overfitting situation. Training and test datasets were overlapping in their work. These gaps were addressed by our proposed HTC-LSTM-Attn model.

Z. Islam et al. [17] forecasted the weather in Bangladesh using a flexible transformer based neural network model pertaining to temperature and rainfall forecasting. Their dataset contains 115 years of monthly based dataset. The authors affirm the strength of the model is lightweight structure and computationally efficient as well as accurate, There were, however, no hierarchical convolutions in their model to catch multi-scale temporal patterns, which our model does extend. The powers of attention in time series forecasting were further substantiated by Bu et al. [18], who established the superiority of attention models over traditional recurrent neural networks in capturing long-range dependencies; by Vaswani et al. [19], who presented the basis for self-attention, now a cornerstone of many modern deep learning architectures.

Deep learning models have been successfully applied for temperature and climate prediction in various regions. Guo et al. (2023) [20] used ANN, LSTM, GRU, CNN, CNN-GRU and CNN-LSTM to predict monthly temperatures in Zhengzhou, China, where CNN-LSTM showed a strong performance. Moreover, Guo et al. (2024a) [21] established the dominance of CNN-LSTM for multi-parameter climate forecasting in Jinan. On the other hand, Guo et al. (2024b) [22] compared multiple DL models for temperature and precipitation in Weifang, highlighting hybrid approaches like CNN-LSTM-GRU.

Elmousaid et al. [23] proposed a hybrid GRU-TCN model for hybrid GRU-TCN model for weather prediction from 2017–2019. This model combined Gated Recurrent Units (GRU) with Temporal Convolutional Networks (TCN) in order to simultaneously capture sequential dependencies and multi-scale temporal patterns. The TCN element is quite similar to what we are applying with our hierarchical temporal convolutions (HTC), but we further enhance its performance through a bidirectional LSTM plus attention mechanism, leading to improved accuracy as validated by our results.

Other literature has applied deep learning for weather forecasting in settings with similar climatic constraints. Thi Kieu Tran, Trang, et al [24] developed an precise time-series prediction model for temperature prediction in India, with a meta-learning for hyperparameter optimization focus. From their work, they found that LSTM has better results in comparison with RNN & ANN, where LSTM had an RMSE of 2.719 °C for temperature prediction, but had difficulty in handling very high variability during monsoon seasons, indicating the urgent requirement for models that can capture multi-scale temporal patterns. Hou, Jingwei, et al. [25] hybrid CNN-LSTM for weather forecasting in China, achieving an MAE of 0.82 °C of temperature. Their model works, yet it does not have an attention mechanism, limiting its capacity to put focus on important time steps, which our model addresses.

A foundational deep learning method underlies our work. Hochreiter and Schmidhuber [26] devised LSTMs that are excellent in capturing long-term dependencies in sequential data, as applied in our bidirectional LSTM component. The core framework for convolutional neural networks is laid down by LeCun et al. [27] and serves itself toward our HTC layers. Goodfellow et al. [13] give a complete overview of the various techniques of deep learning along with the theoretical basis towards the conjunction between convolutional and recurrent architectures as we are doing in the HTC-LSTM-Attn model.

Preprocessing methods also play a major role in the field of model development as far as weather forecasting is concerned. For Bangladesh, Vasenin, Dmitrii, et al. [28] examined such techniques with the emphasis that missing values and seasonal features should be taken into consideration, which is coherent with our pre-processing methods (KNN imputation, seasonal features: Month_sin and Month_cos). The cited work brings arguments on the role of preprocessing methods in enhancing performance at regions in which datasets are noisy or incomplete.

Accurate weather forecasting makes a great difference in primitive Bangladesh, mainly agricultural and disaster management. Rahman et.al [3] researched how variability in weather has affected agricultural productivity in Bangladesh and found that temperature and humidity fluctuations at such times could reduce yields by up to 15% at the most extreme points of weather. Other studies have gone a long way in the field of weather forecasting using deep learning techniques. For instance, Hennayake et al. [29] proposed a multivariate LSTM model to do short-term weather predictions in Sri Lanka, achieving MAE of 0.98985 °F temperature forecast verified. Zaytar et al. [30] implemented multi-stacked LSTM architectures for the weather forecasting model in Morocco using 15 years hourly meteorological data (2000–2015) to predict weather for hours 24 and 72 and proved how LSTM-based neural networks were at par with the traditional approaches. Srivastava et al. [31] showed how LSTM with Gaussian and Median filtering can increase the accuracy of long-range weather predictions; according to them, noise reduction methods were efficient in improving model performance. All the same, such studies generally deal with single-target forecasting without multi-scale temporal characteristics found in weather data from Bangladesh or without the consideration of attention mechanisms related to the important time steps.

Transformers and their derivatives have become the main tools for time series forecasting in recent years due to their ability to manage long-range dependencies and detect patterns at different scales. Among others in their fleet, Autoformer [32] presented decomposition and auto-correlation techniques for long-term predictions. The FEDformer [33] model goes a step further by applying the frequency-domain approach to spot the fluctuations in multivariate data. TimesNet [34] interprets temporal changes as 2D pictures, thus, being capable of supporting the handling of intricate seasonal trends very well. In regard to weather, Local Weather Forecasting [35] suggests using spatiotemporal transformers for high-resolution downscaling, which highlights local variations in climate data. Our HTC-LSTM-Attn model takes a cue from these approaches and combines hierarchical convolutions with attention mechanism, hence, achieving better performance than the competitor models in making multi-target monthly forecasts for the climate of Bangladesh. While not directly applied to meteorology, recent advances in environmental pattern recognition—e.g., in optical transmission under dynamic atmospheric conditions [36] —highlight the importance of feature robustness to regime shifts, a principle we embed via multi-scale convolutions and attention.

Our work builds on these efforts by introducing the HTC-LSTM-Attn model, which integrates hierarchical temporal convolutions to capture multi-scale temporal patterns, bidirectional LSTMs to model sequential dependencies, and an attention mechanism to focus on critical time steps. This approach addresses the limitations of previous models and aims to improve the accuracy of multi-target weather forecasting in Bangladesh, as demonstrated through both numerical metrics and visual comparisons across 24 stations such as Dhaka, Chittagong, and Sylhet etc.

## 3 Dataset

The weather data set includes records from 1961 to 2022. Weather data were collected from the Central Climate Information Management System, Bangladesh Agricultural Research Council (BARC) [14]. The dataset contains monthly maximum temperature, rainfall, humidity, wind speed, cloud cover, and sunshine data for 33 weather stations across Bangladesh. The measurement covers a large geographical range and forms a good diversity of weather patterns due to monsoon effects, river systems, hill track and coastal influences across the region. As a provision for context about the climate of Bangladesh, the Bangladesh Bureau of Statistics (BBS) states that the country has average annual temperatures between 25 and 35 degrees Celsius and that humidity often exceeds 80% during the monsoon [2].

### 3.1 Data pre-processing

Generally, this preprocessed data is confirmed as high-quality and reliable, making it ready for the HTC-LSTM-Attn model. Impurities in the data affect the performance and generalization of the model very significantly [28]. Preprocessing steps included data consolidation, cleaning and enhancement to provide a final dataset defined as “organized_Weather_Data,” ready for deep learning-based weather forecasting. A detail explanation is given in S2 Appendix.

#### 3.1.1 Integrated data.

We considered all the CSV files and merged into a single DataFrame by using Station Code, Year (1961–2022), and Month (1–12) which are the keys. A detail explanation is given in S2 Appendix.

#### 3.1.2 Removal of flat stations.

For data quality, we excluded 11 stations due to their lack of data which were showing minimal variation or high discrepancies. They failed to capture dynamic patterns for weather which is a requirement for forecasting in Bangladesh due to her monsoon-driven climate [1]. The other 24 stations such as Dhaka, Chittagong, Sylhet etc. were considered for their regular data and representation of diverse climate regions [2].

#### 3.1.3 Outliers detection and handling.

Outliers, often due to sensor errors or extreme weather, were detected using the Interquartile Range (IQR) method on numerical columns, where values below or above were replaced with the nearest non-outlier value within the same station to maintain continuity [13,28].

#### 3.1.4 Missing data handling.

Due to sensor issues or different historical gaps, missing values are common in time-series weather data set. This can be overcome using K-Nearest Neighbors (KNN) with k = 5 with leveraging Scikit-learn’s efficient implementation to scale for data [28,37,38]. K-nearest neighbor algorithm can predict missing values by finding out the nearest five data points.

#### 3.1.5 Feature engineering on temporal patterns.

We added seasonal features (e.g., month, season) and lagged features (3-month and 12-month lags for temperature and humidity) to capture temporal dependencies, along with rolling statistics (e.g., 3-month and 12-month moving averages) for key variables, inspired by prior work on different seasons and climates [24,25].

#### 3.1.6 Feature selection.

The data was run through a Pearson’s correlation analysis, and features that were highly correlated (correlation > 0.9) were discarded to avoid multicollinearity, which decreases the dimensionality of the dataset, rather than the predictive capabilities of the HTC-LSTM-Attn model [13,29].

#### 3.1.7 Data normalization.

The numerical features were normalized using min-max scaling to scale them to the range of [0, 1], with parameters for scaling computed on the training set and later applied on the test set in order to preclude data leakage [11,13,39].

#### 3.1.8 Data splitting.

To ensure robustness and eliminate data leakage, especially taking into consideration the diverse character of meteorological data- we implement a rigorous spatio-temporal division in a manner similar to the large-scale forecasting benchmarks [15]. The weather data from the 24 different stations are thus divided at the following time periods:

- Training set (19 stations): 1961–2012
- Validation set (same 19 stations): 2013–2015 (used for hyperparameter tuning and early stopping)
- Temporal test set (same 19 stations): 2016–2022
- Spatial test set (5 held-out stations): 5 completely unseen stations[12007 (Bogra), 10120 (Dinajpur), 10724 (Srimangal), 11513 (Madaripur), 11805 (Feni), 12007 (Rangamati)], which were excluded from all training and validation phases; years 2016–2022

**Fig 1:**
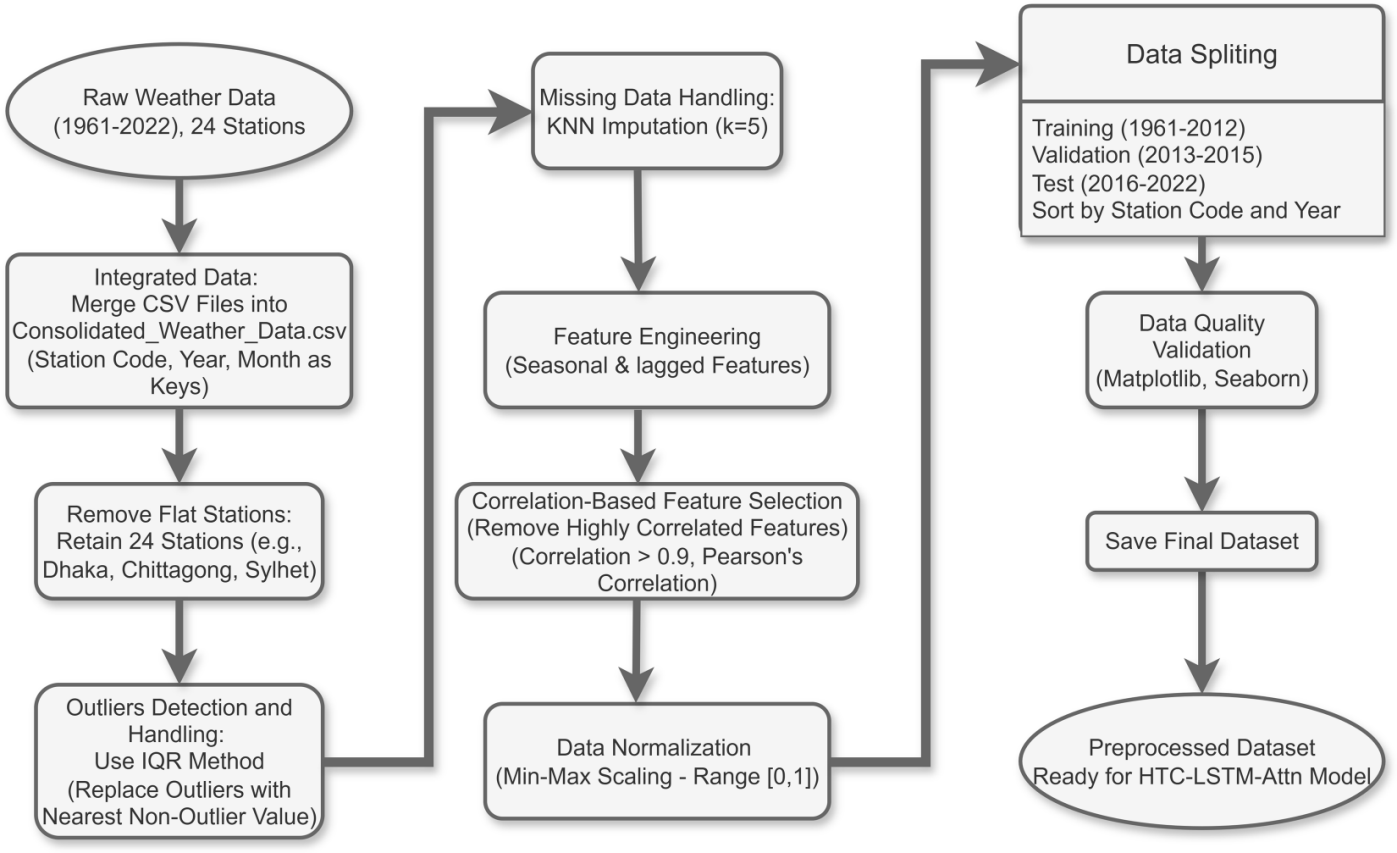
Overview of the data preprocessing pipeline for multi-target weather forecasting. Starting from raw monthly weather data (1961–2022, 24 stations) from the Bangladesh Agricultural Research Council (BARC), the process includes missing data handling via KNN imputation (k = 5), integration of seasonal features (e.g., Month_sin, Month_cos) and lagged statistics (e.g., 1-3 month lags, rolling means/std), outlier detection using IQR with replacement by nearest non-outlier values, correlation-based feature selection (removing highly correlated features with Pearson’s > 0.9), data normalization (Min-Max scaling to [0,1]), and quality validation (e.g., using Matplotlib and Seaborn). The data is split temporally and spatially: training (1961–2012), validation (2013–2015), and test (2016–2022) sets, sorted by station code and year, with exclusion of flat stations (e.g., Tangail, Syedpur, Mongla etc.). This ensures no information leakage and prepares sequential inputs (time steps = 12) for the **HTC-LSTM**-Attn model.

## 4 Methodology

### 4.1 Proposed HTC-LSTM-Attn model

Herein, HTC-LSTM-Attn, a novel deep-learning architecture specially designed for multi-target weather forecasting. The model serves particularly well for the forecasting of weather variables in case of Bangladesh where weather patterns turn out to be complex temporally due to monsoon, seasons, and location-related diversity. The architecture of HTC-LSTM-Attn consists of several key components strategically designed to capture various characteristics. A detailed mathematical formulation of each block is presented subsequently (Fig 1).

We have the HTC-LSTM-Attn model architecture powered by the synergistic integration of LSTMs, attention mechanism, and HTC—a triad that helped multifaceted problems arising in multi-target weather forecasting for Bangladesh. LSTMs, presented by Hochreiter and Schmidhuber [26], exploit memory cells and gating mechanisms (including forget, input, and output gates) to model long-term dependencies in sequential data. This is an effective advantage for weather time series, where seasonal cycles and inter-annual trends may last a few decades, just like the 1961–2022 BARC dataset. The bidirectional version takes this a step further by allowing sequences to be processed both forward and backward, providing valuable context from the past (such as prior dry seasons) and future implications (like potential floods), something paramount in an area of differing climatic influences that include Bangladesh’s monsoon, riverine, and coastal zones.

Standard LSTM processes all time steps uniformly, ignoring the rain-weather connection, especially that some weather events disproportionately affect temperature and humidity, are more important than others. The attention mechanism proposed by Vaswani et al. [19] in the Transformer architecture assigns adaptive weights to the time steps depending on their predictive significance. This way, the model focuses on crucial periods, such as times of sudden temperature anomalies or significant shifts in humidity, enabling better predictions of data characterized by noise and high variance. Unlike pure attention-based models such as the Transformer, which depend entirely on self-attention and thrive in highly parallelized tasks, these may tend toward overfitting or an inefficient exploration of the search space for longer and noisier sequences-natured weather data. The quadratic complexity with length characterizing Transformers [19] contrasts with the linear scalability enjoyed by the LSTMs, thereby making the hybrid approach favorable for handling the outsize dataset of 732 months considered in this study.

The synergy between the LSTM and the attention mechanism is achieved by leveraging their complementary strengths: LSTM provides a robust memory structure essential for retaining long-term temporal context, attention however, takes this further by dynamically weighing what inputs are relevant. This hybrid architecture, combined with HTC layers for multi-scale feature extraction, is hypothesized to outperform stand-alone approaches in balancing sequential modeling and selective attention for the complex weather dynamics of Bangladesh. This theoretical basis supports the novelty and applicability of the architecture-a major gap in previous models such as unidirectional LSTMs [6] or attention-only Transformers [17] -which do not share the temporal hierarchy and memory capacity offered by the present one.

#### 4.1.1 Hierarchical Temporal Convolutional (HTC) layers.

The HTC component comprises three parallel Conv1D layers with kernel sizes of 1, 3, and 5, respectively, to extract multi-scale temporal features from the input sequence $X\in {\mathbb{R}}^{T\times F}$, where *T* is the sequence length (12 months) and *F* is the number of features. For the *k*-th Conv1D layer with kernel size $S_{k}$, the output is:


$$H_{k}=Conv1D(X,W_{k},b_{k})=ReLU(BN(X*W_{k}+b_{k}))$$ (1)

Where $W_{k}\in {\mathbb{R}}^{S_{k}\times F\times N_{k}}$ are the convolution weights ($N_{k}$ is the number of filters: 96 for $S_{k}$ = 1, 64 for $S_{k}$ = 3, 96 for $S_{k}$ = 5) $b_{k}$ is the bias, * denotes the convolution operation, BN is batch normalization, and *ReLU (x) = max (0,x)*. The outputs of the three layers are concatenated:


$$H_{HTC}=Concat\left(H_{1},H_{3},H_{5}\right)$$ (2)

The outputs of the three parallel convolutional branches (kernel sizes 1, 3, and 5 with 96, 64, and 96 filters respectively) are concatenated along the channel dimension, yielding $H_{HTC}$ of shape (batch_size, 12, 256). A feature combination Conv1D layer with kernel size 3 and 128 filters is applied:


$$H_{combined}=Conv1D(H_{HTC},W_{comb},b_{comb})$$ (3)

followed by batch normalization, ReLU activation, and a dropout layer with a rate of 0.1 to prevent overfitting. This produces $H_{combined}$ of shape (batch_size, 12, 128), which is subsequently fed to the bidirectional LSTM layer. This approach has been inspired by the successful application of Temporal Convolution Networks (TCNs) as being superior to recurrent neural networks in specific time-series learning tasks, given their ability to learn long-range dependencies with fewer parameters [40]. The HTC layers, therefore, have 96 filters for 1-kernel size, 64 filters for 3-kernel size, and 96 filters for 5-kernel size based on appropriate hyperparameter tuning. The convolutional layers for temporal feature extraction rely on much earlier work in deep learning, such as applying CNNs towards sequence modeling [27], which have gained appreciable attention in diverse areas [13].

#### 4.1.2 Bidirectional LSTM.

A bidirectional LSTM layer with 96 units (i.e., 96 neurons per direction, capturing sequential dependencies) processes $H_{combined}$ to capture sequential dependencies in both forward and backward directions. For a time step *t*, the forward LSTM computes:


$${\vec{h}}_{t}=LSTM\left(H_{combined,t},{\vec{h}}_{t-1},{\vec{c}}_{t-1}\right)$$ (4)

and the backward LSTM computes:


$$\overset{\leftarrow }{h_{t}}=LSTM\left(H_{combined,t},\overset{\leftarrow }{h_{t-1}},\overset{\leftarrow }{c_{t-1}}\right)$$ (5)

The LSTM cell, as introduced by Hochreiter and Schmidhuber, updates are defined as [26]:


$$\left\{ \begin{array}{c} f_{t}=\sigma (W_{f}\cdot [h_{t-1},x_{t}]+b_{f})\\ i_{t}=\sigma (W_{i}\cdot [h_{t-1},x_{t}]+b_{i})\\ o_{t}=\sigma (W_{o}\cdot [h_{t-1},x_{t}]+b_{o}) \end{array}\right.$$ (6)

$$\vec{C_{t}}=\tanh(W_{C}\cdot [h_{t-1},x_{t}]+b_{C})$$ (7)

$$C_{t}=f_{t}\circ C_{t-1}+i_{t}\circ \vec{{\tilde{C}}_{t}}$$ (8)

$$h_{t}=o_{t}\circ \tanh(C_{t})$$ (9)

where *σ* is the sigmoid function, $f_{t}$, $i_{t}$, $o_{t}$, are the forget, input, and output gates, and $C_{t}$ is the cell state with ${\vec{C}}_{t}$ define as cell state candidate, $h_{t}$ denotes as hidden state. The hierarchical temporal convolutional features are processed by a bidirectional LSTM layer with 96 units in each direction. The bidirectional output is:


$$h_{t}=concat\left(\vec{h_{t}},\overset{\leftarrow }{h_{t}}\right)\in {\mathbb{R}}^{2\times 96}$$ (10)

where $\vec{h_{t}}$, $\overset{\leftarrow }{h_{t}}$ are the hidden states of the forward and backward LSTMs respectively, each of dimension 96. A dropout layer with a rate of 0.1 is applied to prevent overfitting. This model is based on the basics of LSTM work and widened to forecasting sequences by LSTM [26] with some practical context as applied to weather fore [41].

#### 4.1.3 Attention mechanism.

The attention mechanism focuses on the most relevant time steps in the sequence. For each time step *t,* attention scores are computed [42]:


$$e_{t}=Dense(h_{t},W_{attn},b_{attn})=\tanh(W_{attn}.h_{t}+b_{attn})$$ (11)

Attention weights are obtained via softmax:


$$\alpha_{t}=\frac{\exp(e_{t})}{\sum_{t^{\prime }=1}^{T}\exp(e_{t^{\prime }})}$$ (12)

The context vector is a weighted sum of the LSTM outputs:


$$c=\sum_{t=1}^{T}\alpha_{t}h_{t}$$ (13)

Inspired by Vaswani et al. [19], this enables the model to focus on time steps that prominently affect the observation of maximum temperature and humidity such as previous weather tendencies and seasonal trends. The use of attention mechanisms in the time series forecasting area has been highly researched and applied to improve the performance of forecast models in identifying relevant temporal features [18]. Also, as seen in previous work on self-attention mechanisms, attests further to the effectiveness of this in deep learning models [43].

#### 4.1.4 Dense layers and prediction.

The context vector *c* is passed through a dense layer with 256 units (i.e., 256 neurons, learning high-level features):


$$z=ReLU(W_{dense}.c+{𝐛}_{dense})$$ (14)

followed by a dropout layer with a rate of 0.5. Two separate dense layers, each with 1 unit, predict the maximum temperature and humidity:


$${\hat{y}}_{temp}=W_{temp}.z+b_{temp}$$ (15)

$${\hat{y}}_{hum}=W_{hum}.z+b_{hum}$$ (16)

**Fig 2:**
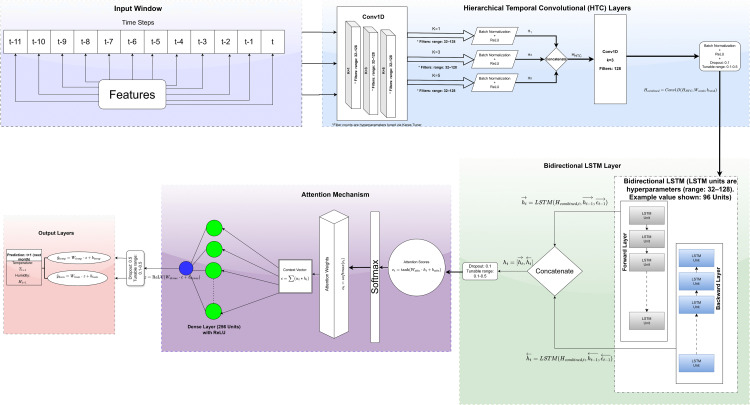
Architecture of the proposed Hierarchical Temporal Convolutional Long Short-Term Memory with Attention (HTC-LSTM-Attn) model for one-month-ahead forecasting of maximum temperature and humidity. The input window consists of a sequence of 12 time steps with multivariate features (e.g., solar radiation, PET, sunshine hours, wind speed, cloud coverage, rainfall, and seasonal/lagged components). Hierarchical Temporal Convolutional (HTC) layers extract multi-scale patterns using Conv1D filters (e.g., 96, 64, 96 filters with kernels k=1,3,5), followed by batch normalization, ReLU activation, and concatenation. Bidirectional LSTM layers (96 units) capture forward and backward sequential dependencies, with an attention mechanism computing context vectors, softmax-weighted scores (e.g., $\alpha_{t}=softmax(e_{t})$), and concatenated hidden states using $h_{t}=LSTM(H_{combined,t},\;h_{t-1},\;c_{t-1})$. The output layers include dense units (256) with ReLU, dropout (0.1), and final predictions for temperature ($T_{t+1}$) and humidity ($H_{t+1}$). This design uses purely historical temporal data up to month *t* to predict the next month (horizon = 1), ensuring no future data leakage.

The detailed architecture of the HTC-LSTM-Attn model, including the flow of data through each layer and the tuned hyperparameters, is illustrated in Fig 2

### 4.2 Training setup and hyperparameter tuning

Hyperparameters that boost the performance of the HTC-LSTM-Attn model are sought using RandomSearch in Keras Tuner. It involves hyperparameter tuning for five trials, minimizing the validation loss (Mean Squared Error, MSE) at each stage. The hyperparameters being searched in this regard are given herewith:

- HTC layer filter numbers: 32–128, where step = 32
- Feature combination Conv1D layer filter numbers: 64–256, where step = 64
- LSTM unit count: 32–128, where step = 32
- Dense layer unit count: 64–256, where step = 64
- Dropout rates for the three respective dropout layers: 0.1 to 0.5, where step = 0.1

All tuning is done on the training and validation sets with early stopping set with patience of 10 to restrict overfitting. The final fine-tuned hyperparameters were set to:

- HTC Filters: 96 with kernel size = 1, 64 with kernel size = 3, 96 with kernel size = 5
- Conv1D Filters feature combination: 128
- LSTM Units: 96
- Dense Units: 256
- Dropout Rates: 0.1 after HTC, 0.1 after LSTM, and 0.5 after Dense

**Fig 3:**
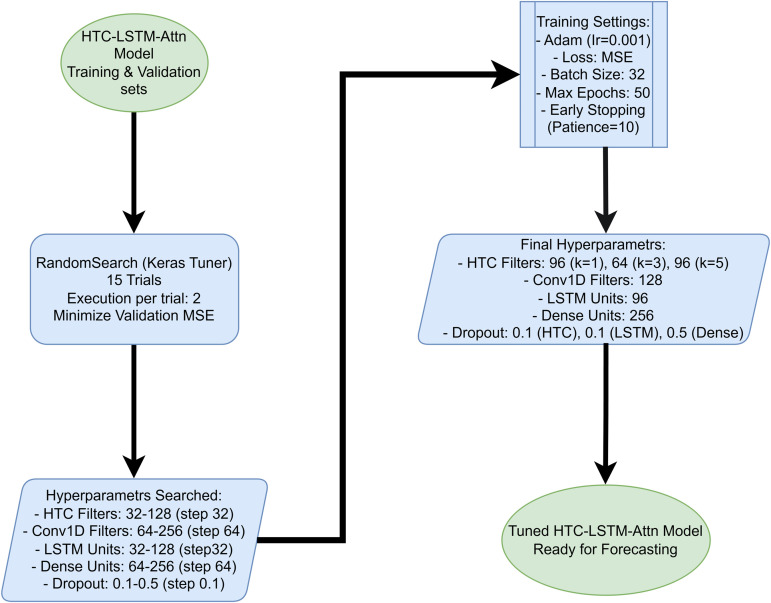
Hyperparameter tuning process using Keras Tuner for the HTC-LSTM-Attn model. Using the training and validation sets (Adam optimizer, MSE loss, batch size 32, maximum 50 epochs, patience 10), a RandomSearch tuner performs 15 trials (2 executions per trial) to minimize validation MSE. The search optimizes key parameters including HTC filters (96 at k=1, 64 at k=3, 96 at k=5), Conv1D filters (128), LSTM units (96), dense units (256), and dropout rates (0.1 for HTC/LSTM and 0.5 for dense). The tuned hyperparameters are then applied to build the final HTC-LSTM-Attn model for forecasting, ensuring robustness across both temporal and spatial test sets while preventing overfitting.

To train the model, the Adam optimizer has been used with a learning rate of 0.001, which has been shown to be effective for training deep learning models [44], the loss function as MSE with the batch size set to 32. This training took place for a maximum of 50 epochs with early stopping in place to stop the training once the validation loss did not improve for 10 consecutive epochs.All baseline models received identical preprocessing, hyperparameter optimization, and training protocol using the same computational resources to ensure fair comparison (Fig 3).

### 4.3 Fair comparative evaluation protocol

Our comparative analysis was based on rigorous methodology, and hence all baseline models (transformer-based models included) went through the same preprocessing, hyperparameter optimization, and training. The details of the same are as under:

- Data preparation: Every model did the same feature engineering (seasonal features, lagged statistics), using the same method to impute missing values (KNN with k = 5) and applied the same data splitting method (temporal: training 1961–2012, validation 2013–2015, testing 2016–2022; spatial: 5 completely unseen stations).
- Hyperparameter optimization: Keras Tuner’s RandomSearch was used to find the best hyperparameters for all the deep learning models with the same search space (e.g., layer units: 32−128, dropout: 0.1–0.5) and tuning budgets (15 trials × 2 executions). Transformer models (Autoformer, FEDformer, TimesNet, and Pyraformer) were given the same optimization resources as our HTC-LSTM-Attn model.
- Training protocol: The same batch size (32), optimizer (Adam with lr = 1e-3), loss function (MSE), early stopping criteria (patience = 10), and evaluation metrics were used by all models.

## 5 Results and discussion

The HTC-LSTM-Attn model has been analyzed and tested for maximum temperature and humidity forecasting over 24 valid stations across Bangladesh, including Dhaka, Chittagong, Sylhet, and others from 2016 to 2022. The performance is compared with the state of the art models in that area, i.e., LSTM, GRU and Conv1D for [6], DTR and CatBoost for [1], CNN-LSTM [25], GRU-TCN [27] and ANN. The performance metrics used for evaluation are Mean Absolute Error (MAE), Root mean squared error (RMSE), R-squared (R²), and Mean Absolute Percentage Error (MAPE), all defined as follows:

**Mean Absolute Error (MAE)** $=\frac{1}{n}\sum_{i=1}^{n}|y_{i}-{\hat{y}}_{i}|$

Root Mean Squared Error (RMSE) $=\sqrt{\frac{1}{n}\sum_{i=1}^{n}(y_{i}-{\hat{y}}_{i}{)}^{2}}$

R-squared (R²) $=1-\frac{\sum_{i=1}^{n}(y_{i}-{\hat{y}}_{i}{)}^{2}}{\sum_{i=1}^{n}(y_{i}-\bar{y}{)}^{2}}$

Mean Absolute Percentage Error (MAPE) $=\frac{1}{n}\sum_{i=1}^{n}\left|\frac{y_{i}-{\hat{y}}_{i}}{y_{i}}\right|\times 100\%$

Where, $y_{i}$ = Actual value, ${\hat{y}}_{i}$= Predicted value, and *n* = Number of observations, $\bar{y}=\frac{1}{n}\sum_{i=1}^{n}y_{i}$ is the mean of the actual values A small epsilon ($\epsilon =10^{-10}$) is added to the denominator in practice to avoid division by zero.

To delineate with clarity the model performance comparisons, we include visual representations of temperature and humidity predictions at various stations, as well as an overall comparison, within the performance metrics. The full set of station-level metrics for all 19 stations for temporal generalization and 5 unseen stations for spatial generalization are given in the **S1 Appendix**.

### 5.1 Performance Metrics

The overall evaluation metrics for the HTC-LSTM-Attn model across all valid stations, as obtained from the evaluation report, are as follows (Tables 1 and 2):

**Table 1:** Overall station-level metrics for the HTC-LSTM-Attn model (Temporal Test).

Prediction Performance Metrics				
Type	MAE	RMSE	R^2^	MAPE
Temperature	0.8178 °C	0.9718 °C	0.8527	2.8823%
Humidity	2.4693%	3.2442%	0.7228	3.1757%

MAE: Mean Absolute Error; RMSE: Root Mean Squared Error; R^2^: Coefficient of Determination; MAPE: Mean Absolute Percentage Error. All values represent average performance across test datasets.

**Table 2:** Overall station-level metrics for the HTC-LSTM-Attn model (Spatial Test: 5 unseen stations).

Prediction Performance Metrics				
Type	MAE	RMSE	R^2^	MAPE
Temperature	0.9587 °C	1.1898 °C	0.8342	3.1534%
Humidity	2.4796%	3.2119%	0.6561	3.2045%

MAE: Mean Absolute Error; RMSE: Root Mean Squared Error; R^2^: Coefficient of Determination; MAPE: Mean Absolute Percentage Error. All values represent average performance across test datasets.

To indicate a particular distinction of the model’s performance at the station level, Table 3 present MAE, RMSE, R², and MAPE for temperature and humidity predictions in five selected stations, namely, Dhaka, Chittagong, Sylhet, Rangpur, and Khulna for the temporal test, whereas the spatial test (Table 4) has been done for the stations named Dinajpur, Srimangal, Madaripur, Feni, and Rangamati. These stations are major centers, each with a different climate, namely the central, south-eastern, north-eastern, northern, and south-western regions of Bangladesh.

**Table 3:** Selected station-level metrics for the HTC-LSTM-Attn model (Temporal).

Area	Temperature				Humidity			
	MAE (°C)	RMSE (°C)	$R^{2}$	MAPE (%)	MAE (%)	RMSE (%)	$R^{2}$	MAPE (%)
Rangpur	1.1114709	1.4125628	0.8137935	3.8161687	2.6970362	3.3070550	0.6781429	3.3938372
Sylhet	1.0713364	1.4277754	0.7782162	3.5498919	3.0834894	3.6714800	0.7424750	3.9716631
Dhaka	0.9334865	1.1623638	0.8541125	3.0782448	3.3947765	4.0676876	0.7464639	4.7850829
Khulna	0.7859715	0.9398312	0.9172091	2.5788885	2.0861848	2.6152242	0.7588976	2.7036831
Chittagong	0.8456525	1.0158162	0.8056897	2.7646010	2.5123626	3.2008108	0.7636111	3.3135914

MAE: Mean Absolute Error; RMSE: Root Mean Squared Error; $R^{2}$: Coefficient of Determination; MAPE: Mean Absolute Percentage Error. Metrics shown for both temperature (in °C) and humidity (in %) predictions across five Bangladeshi regions under the temporal setting.

**Table 4:** Selected station-level metrics for the HTC-LSTM-Attn model (Spatial).

Area	Temperature				Humidity			
	MAE (°C)	RMSE (°C)	$R^{2}$	MAPE (%)	MAE (%)	RMSE (%)	$R^{2}$	MAPE (%)
Dinajpur	1.1812344	1.5030673	0.8155824	4.0789336	2.4462002	3.0081912	0.6403405	3.1052416
Srimangal	1.0007081	1.1971783	0.8209514	3.2603860	2.3012758	2.8314229	0.6353528	2.8277746
Madaripur	0.7845270	0.9332649	0.9115502	2.5539153	1.6399630	2.2252525	0.7763504	2.0658090
Feni	0.9437520	1.1557389	0.7923330	3.0523552	2.4923770	3.1232502	0.6724410	3.2745455
Rangamati	0.9607177	1.1773816	0.7997133	3.0839687	3.7300585	4.6764973	0.5956969	5.0782362

MAE: Mean Absolute Error; RMSE: Root Mean Squared Error; $R^{2}$: Coefficient of Determination; MAPE: Mean Absolute Percentage Error. Metrics shown for both temperature (in °C) and humidity (in %) predictions across five Bangladeshi regions under the spatial setting.

The **S1 Appendix** indicates comprehensive metrics across all 19 stations for temporal generalization. The performance of the station-level analysis is consistent across different regions for temporal test, where the MAE for temperature is 0.6980 °C (Satkhira) to 1.11 °C (Rangpur), while the humidity MAPE is found between 1.9368% (Satkhira) and 3.5767% (Sitakunda).

For the maximum temperature forecasting using the HTC-LSTM-Attn model, we acquire MAE of 0.8178 °C, RMSE of 0.9718 °C, R² of 0.8527, and MAPE of 2.8823% for temporal test and for spatial generalization we got, MAE of 0.9587 °C, RMSE of 1.1898 °C, R² of 0.8342, and MAPE of 3.1534% which outpaced other deep learning model like ANN,KNN, LSTM, GRU,Conv1D, Hybrid model CNN-LSTM and SOTA models. From Table 5, our model shows better result compared to other studies. Our HTC-LSTM-Attn model outperforms the results reported by Mumu et al. [6] i.e, their LSTM (MAE: 1.68 °C, RMSE: 2.15°C, MAPE: 5.38%), GRU (MAE: 2.17 °C, RMSE: 2.64°C, MAPE: 6.95%), and Conv1D (MAE: 1.75 °C,RMSE: 2.21 °C MAPE: 5.59%) models. Compared to the ensemble ML models from Mahabub et al. [1], our model with MAE of 0.8178 °C falls well short of what is likely unrealistic and over-fit Decision Tree Regressor (DTR)-amendable MAE of 0.0 °C (MAPE: 0.0%) given the rarity of such low error in real-world weather forecasting tasks with intrinsic noise and variability. But they achieved a more reasonable and almost the same results for their KNN (MAE: 0.785 °C, RMSE: 0.97 °C, MAPE: 2,65%) model. But they did not split their dataset into training, validation and test datasets; rather, they split their dataset only into training and test datasets which may lead to overfitting due to the overlapping datasets. Besides these, from Hou et al. [25], we got, MAE for CNN is 1.38, CNN-LSTM is 1.02 and LSTM is 1.29. Additionally, for RMSE they got the results for CNN,CNN-LSTM, LSTM are 3.14,0.8,2.72 respectively. Also, R² is much lower than our model. Moreover, we have run state-of-the-art models on our datasets [45], where Pyformer, Autoformer, FEDFormer and TimesNet all showed lower performance in comparison with our novel model named HTC-LSTM-Attn for both temporal and spatial tests. For this, this indicates the way the HTC-LSTM-Attn model beat the others in reference to the tricky weather conditions in Bangladesh, which shows a robust result.

**Table 5:** Performance comparison for maximum temperature forecasting.

Model	Type	MAE (°C)	RMSE (°C)	R^2^	MAPE (%)
HTC-LSTM-Attn (Novel Model)	Temporal	0.8178	0.9718	0.8527	2.8823
	Spatial	0.9587	1.1898	0.8342	3.1534
ANN	–	1.5803	1.9557	0.4219	5.1492
LSTM [6]	–	1.68	2.15	–	5.38
GRU [6]	–	2.17	2.64	–	6.95
Conv1D [6]	–	1.75	2.21	–	5.59
DTR [1]	–	0.0	0.0	–	0.0
KNN [1]	–	0.785	0.97	–	2.65
CNN [25]	–	1.38	3.14	0.735	–
CNN-LSTM [25]	–	1.02	0.8	0.5787	–
LSTM [25]	–	1.29	2.72	0.52	–
CatBoost [1]	–	–	–	–	36.93
Pyform	Temporal	0.9559	1.2112	0.8211	3.1333
	Spatial	1.1566	1.4327	0.7841	3.8610
Autoformer	Temporal	1.0117	1.3146	0.7892	3.2736
	Spatial	1.1253	1.3932	0.7958	3.7029
FEDformer	Temporal	2.3881	2.8403	0.0163	7.9291
	Spatial	2.4298	2.9864	0.0619	8.2488
TimesNet	Temporal	0.9856	1.2061	0.8226	3.1819
	Spatial	1.0757	1.3491	0.8085	3.5520

MAE: Mean Absolute Error; RMSE: Root Mean Squared Error; R^2^: Coefficient of Determination; MAPE: Mean Absolute Percentage Error. Dashes (- or –) indicate unavailable metrics. Temporal and spatial denote forecasting modes for respective models. Reference numbers in brackets correspond to citations.

For the humidity forecasting using the HTC-LSTM-Attn model (Table 6), we get MAE of 2.4693%, RMSE of 3.2442%, R² of 0.72281, and MAPE of 3.1757% from the temporal test, on the other hand, from spatial test, we get MAE of 2.4796%, RMSE of 3.2119%, R² of 0.6561, and MAPE of 3.2045%, again trumping over other deep learning model which are tested by Mumu et. Al [6] like LSTM, GRU, Conv1D where MAE for these models are 4.34%, 4.56%, 3.47% respectively. Additionally, RMSE obtained for these models (LSTM, GRU, Conv1D) are 5.40%, 5.78%, 4.24%. For them we got MAPE performances are 6.23%, 6.70%, 4.88% correspondingly. Additionally, ANN has lower performance evaluation in comparison with HTC-LTM-Attn model in every performance metrics. However, Mahabub et al. [1] did not analyze humid forecast to compare this direct observation with their ensemble ML models on this target. Additionally, we have run state-of-the-art models on our datasets [45], where Pyformer, Autoformer, FEDFormer and TimesNet all showed lower performance in comparison with our novel model named HTC-LSTM-Attn for both temporal and spatial tests. This indicates that our approach not only outperforms existing models but also offers a more robust result for managing complex temporal and spatial data challenges.

**Table 6:** Performance comparison for humidity forecasting models.

Model	Type	MAE (%)	RMSE (%)	R^2^	MAPE (%)
HTC-LSTM-Attn (Novel Model)	Temporal	2.4693	3.2442	0.7228	3.1757
	Spatial	2.4796	3.2119	0.6561	3.2045
ANN	–	3.9590	5.0402	0.4318	5.5435
LSTM [6]	–	4.34	5.40	–	6.23
GRU [6]	–	4.56	5.78	–	6.70
Conv1D [6]	–	3.47	4.24	–	4.88
Pyform	Temporal	2.7021	3.5876	0.6449	3.4734
	Spatial	3.4936	4.5453	0.3308	4.6436
Autoformer	Temporal	2.8796	3.9749	0.5641	3.7224
	Spatial	5.0641	6.3824	0.3193	6.8132
FEDformer	Temporal	4.7068	5.7403	0.0909	5.9850
	Spatial	4.2922	5.3671	0.0670	5.6919
TimesNet	Temporal	2.4691	3.2256	0.7129	3.1456
	Spatial	3.1328	3.9444	0.4961	4.1395

MAE: Mean Absolute Error; RMSE: Root Mean Squared Error; R^2^: Coefficient of Determination; MAPE: Mean Absolute Percentage Error. Dashes (–) denote unavailable metrics. Temporal and spatial indicate forecasting modes for respective models.

### 5.2 Visual comparisons across stations

More evidence of the HTC-LSTM-Attn model performance will be shown using the visual comparisons of the temperature and humidity simulations over the various stations in Bangladesh. These will include comparisons of HTC-LSTM-Attn with baselines such as CNN, LSTM, GRU, and ANN, as well as SOTA models like Pyformer, Autoformer, FEDFormer and TimesNet. The actual counter-parts of the predicted values across time will be shown for the selected stations (for temporal): Dhaka, Chittagong, Sylhet, Rangpur, and Khulna; for spatial: Rangamati and Dinajpur. These stations represent the most important urban centers in Bangladesh with different climatic zones, serving the whole of central, southeastern, northeastern, northern, and southwestern regions.

**Fig 4:**
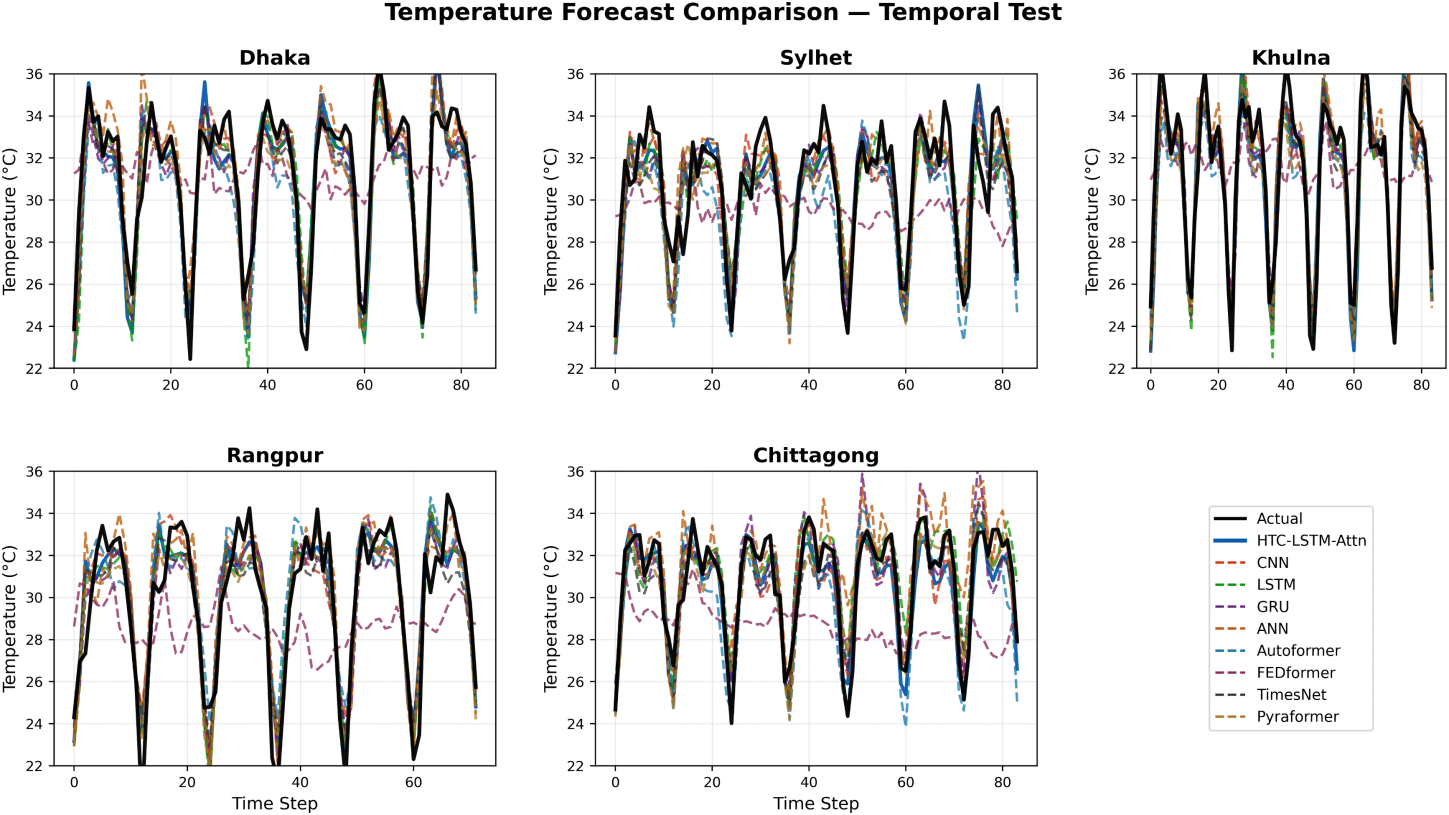
Temperature prediction comparisons for selected stations (Temporal).

**Fig 5:**
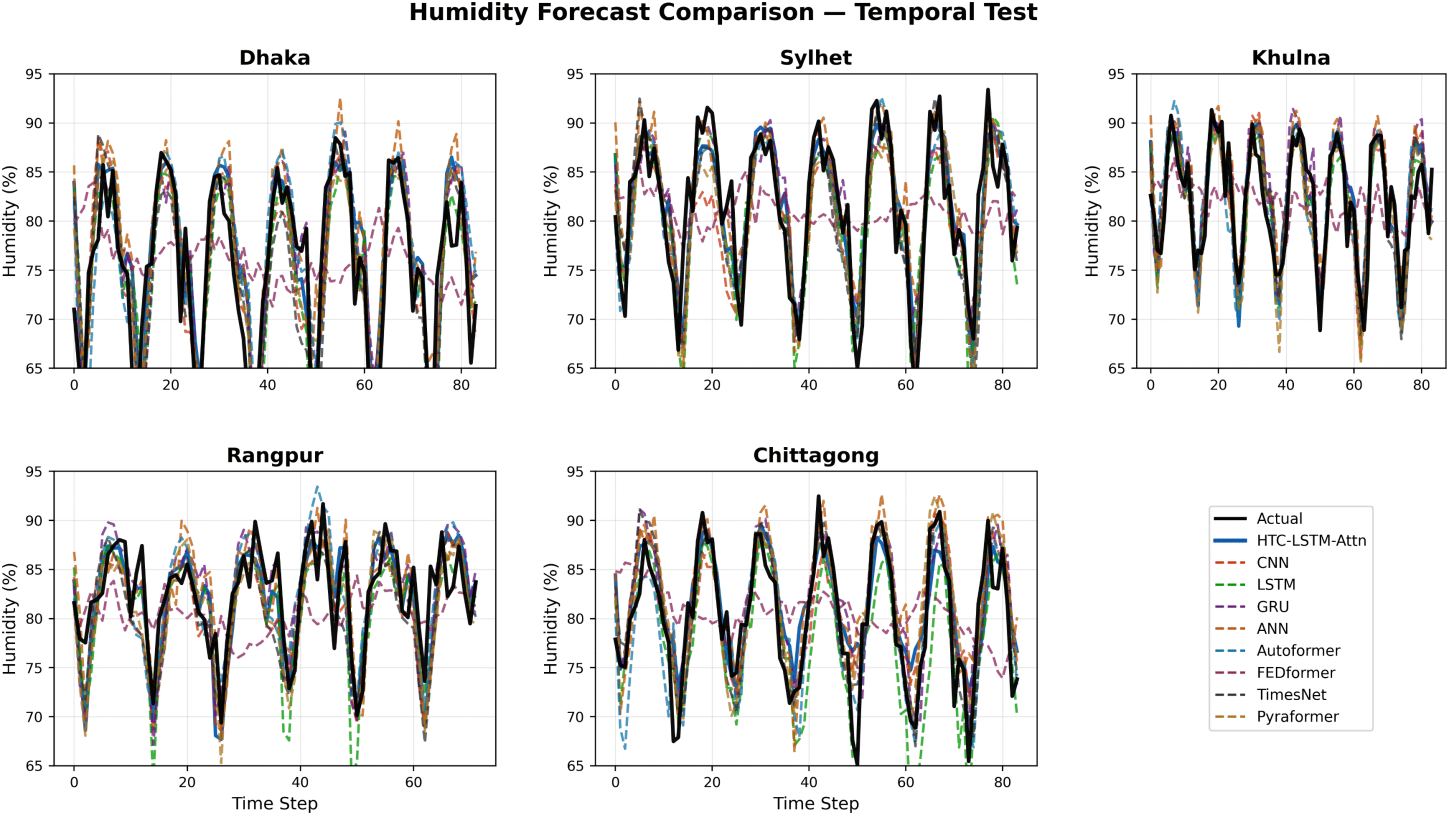
Humidity prediction comparisons for selected stations (Temporal).

**Fig 6:**
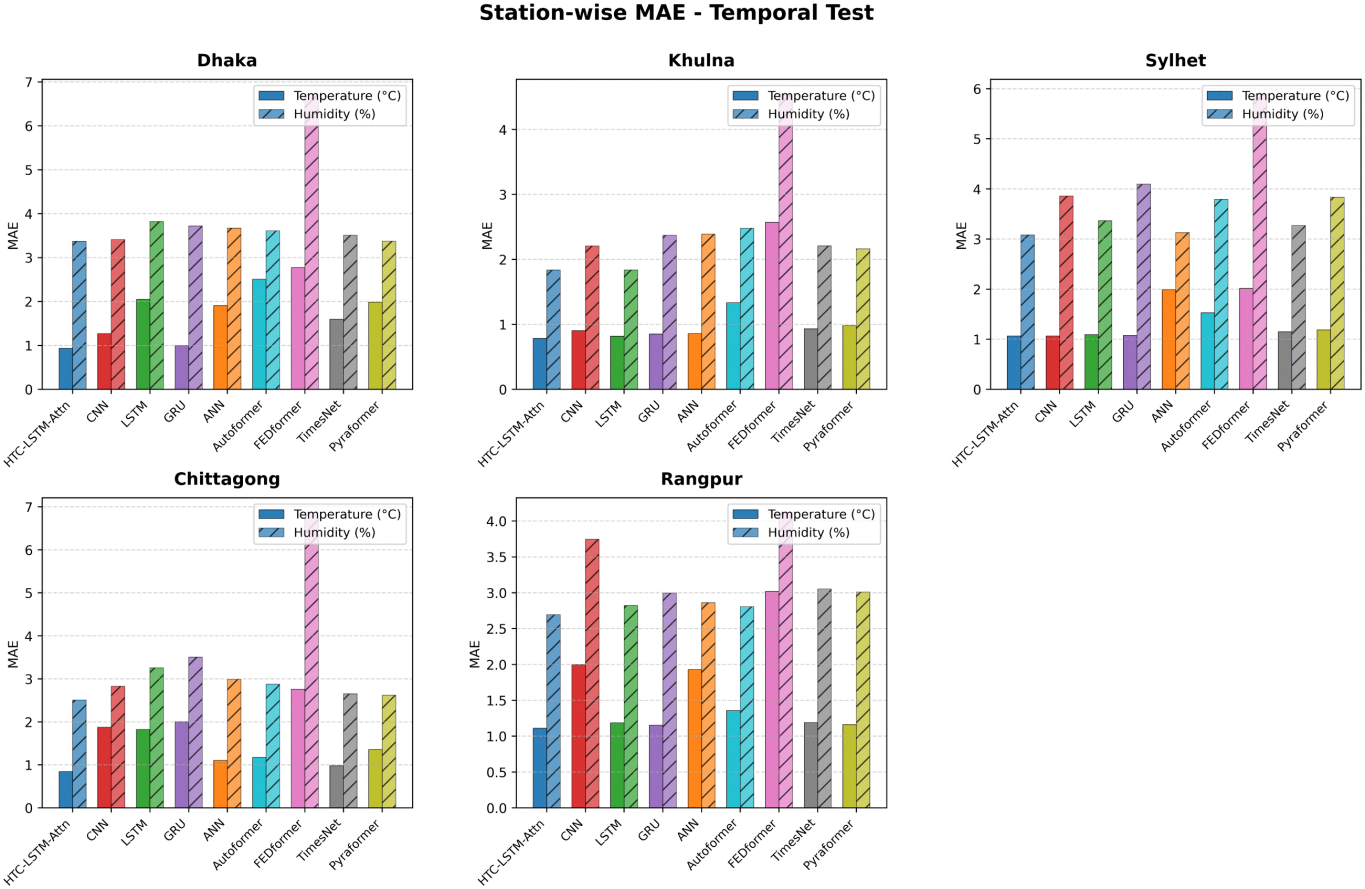
Prediction (Temperature & Humidity) comparisons for selected stations (Temporal)-Bar Chart.

**Fig 7:**
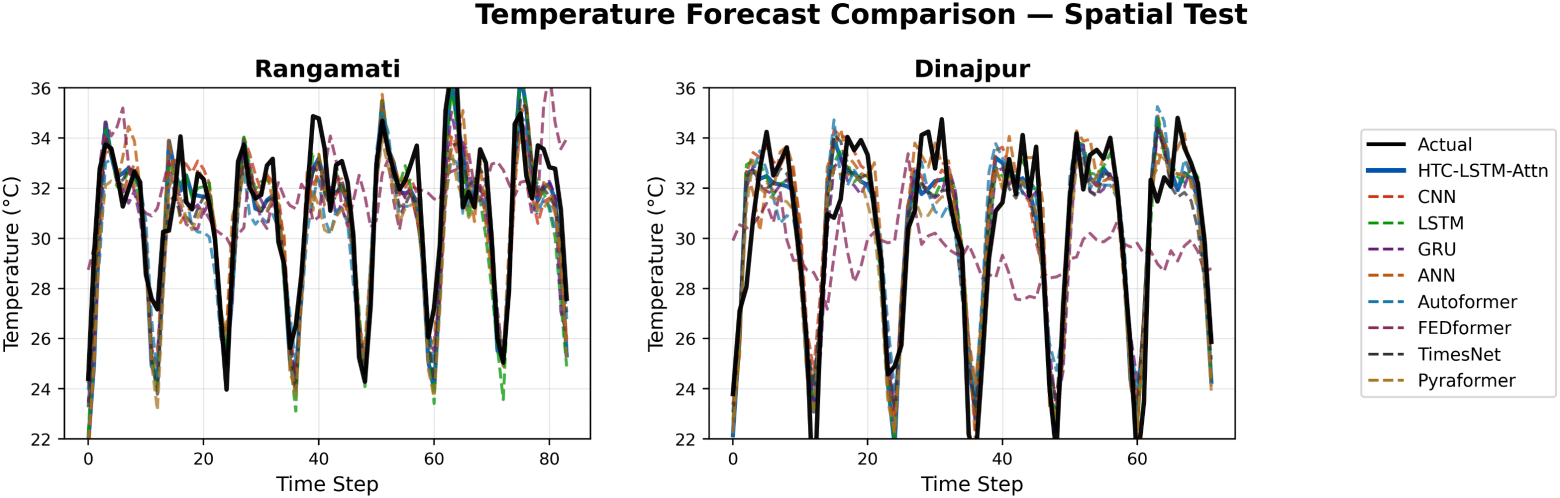
Temperature prediction comparisons for selected stations (spatial).

**Fig 8:**
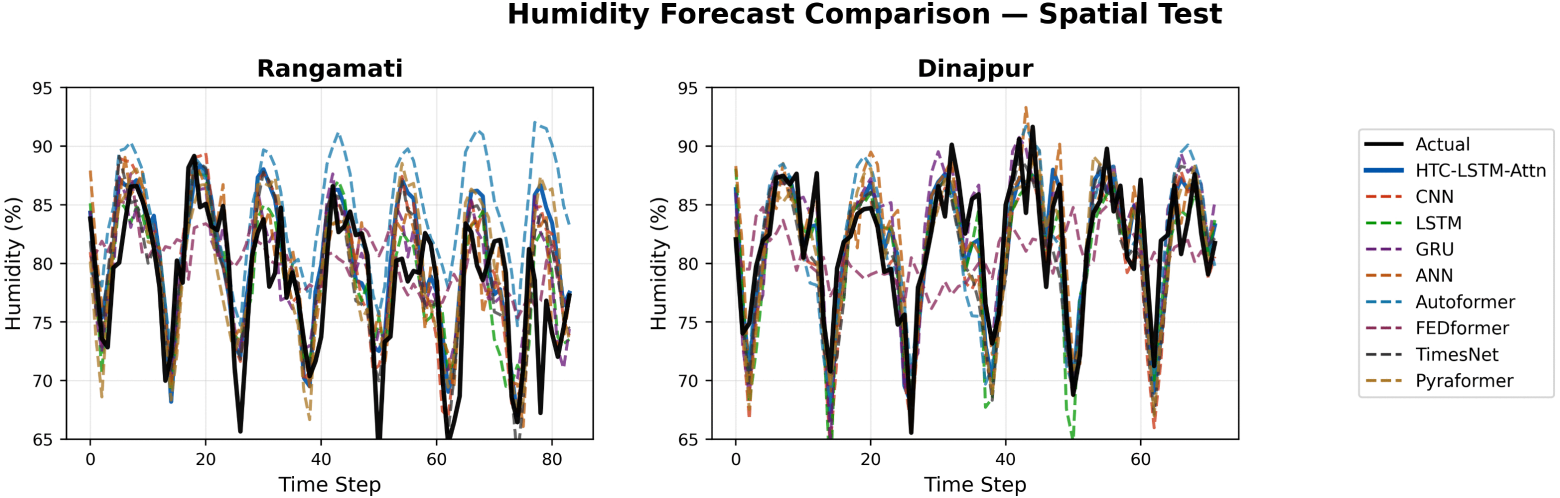
Humidity prediction comparisons for selected stations (Spatial).

**Fig 9:**
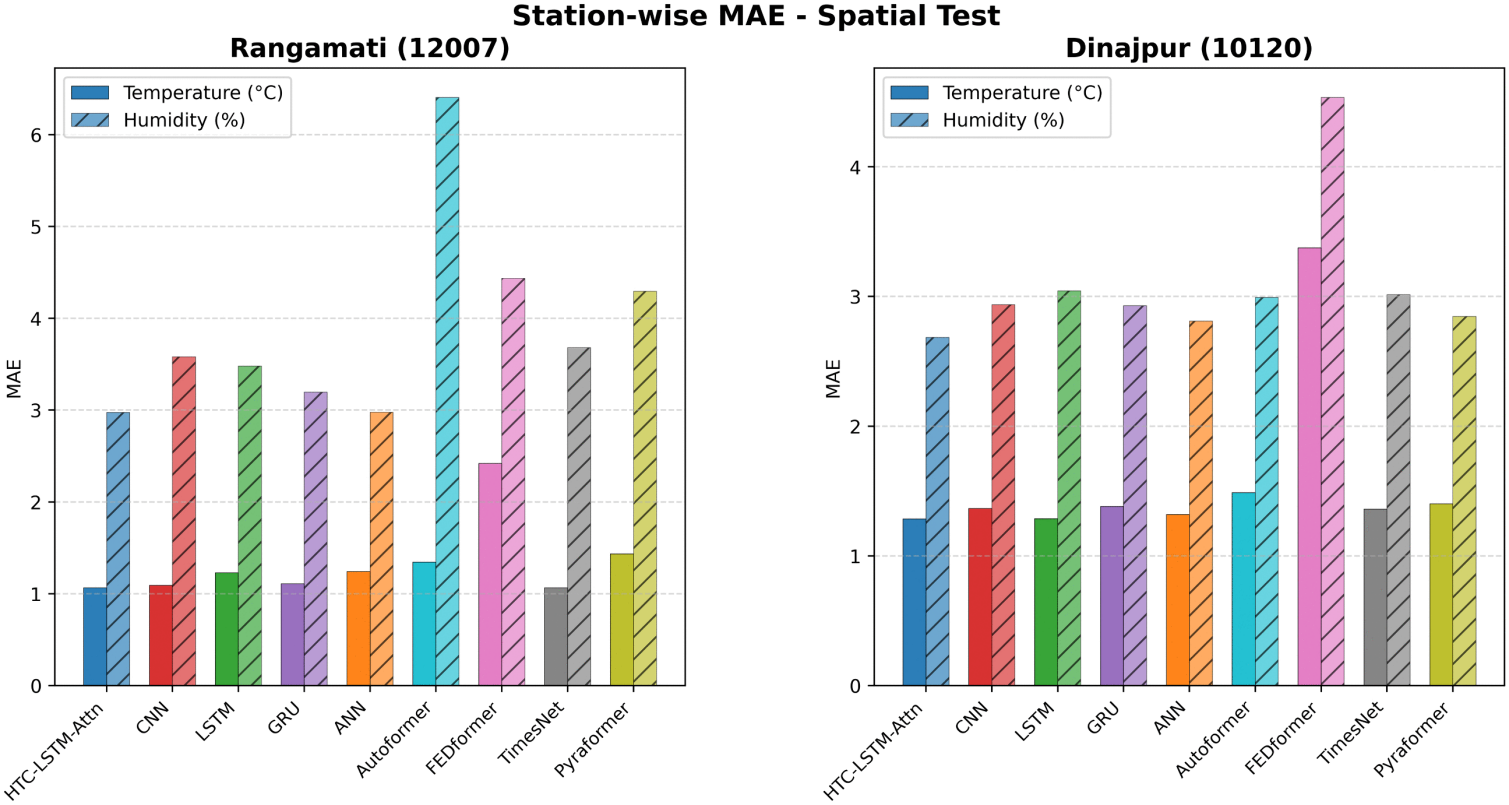
Prediction (Temperature & Humidity) comparisons for selected stations (Spatial)-Bar Chart.

Figs 4–9 depict how the HTC-LSTM-Attn model can no longer yield predictions that would show the actual temperature and humidity values at all the selected stations for temporal and spatial generalization. For example, while looking at two stations like Dhaka and Chittagong, it has been discovered that this model is better at predicting seasonal trends and sudden temperature changes compared to CNN, LSTM, GRU, ANN and SOTA models than the latter, which exhibit a higher disparity with respect to the actual values. In Sylhet, with a high monsoon variability, it has shown that the changing pattern of atmospheric humidity has successfully been followed. Again, in Rangpur and Khulna, which show the northern and southwestern climate types, the same utmost performance continues for the HTC-LSTM-Attn with respect to temperature and humidity. These comparisons are purely visualized so that one can evaluate their capability to generalize concerning diverse geographical regions in Bangladesh, where the same weather does not reflect local climatic conditions.

### 5.3 Overall performance comparison

To provide a comprehensive overview of the HTC-LSTM-Attn model’s performance, we present plots of actual vs. predicted time series for overall temperature and humidity across the 19 stations for temporal generalization and 5 unseen stations for spatial generalization, showing the comparison between the HTC-LSTM-Attn model and the baseline models in terms of overall predictive accuracy across all stations.

**Fig 10:**
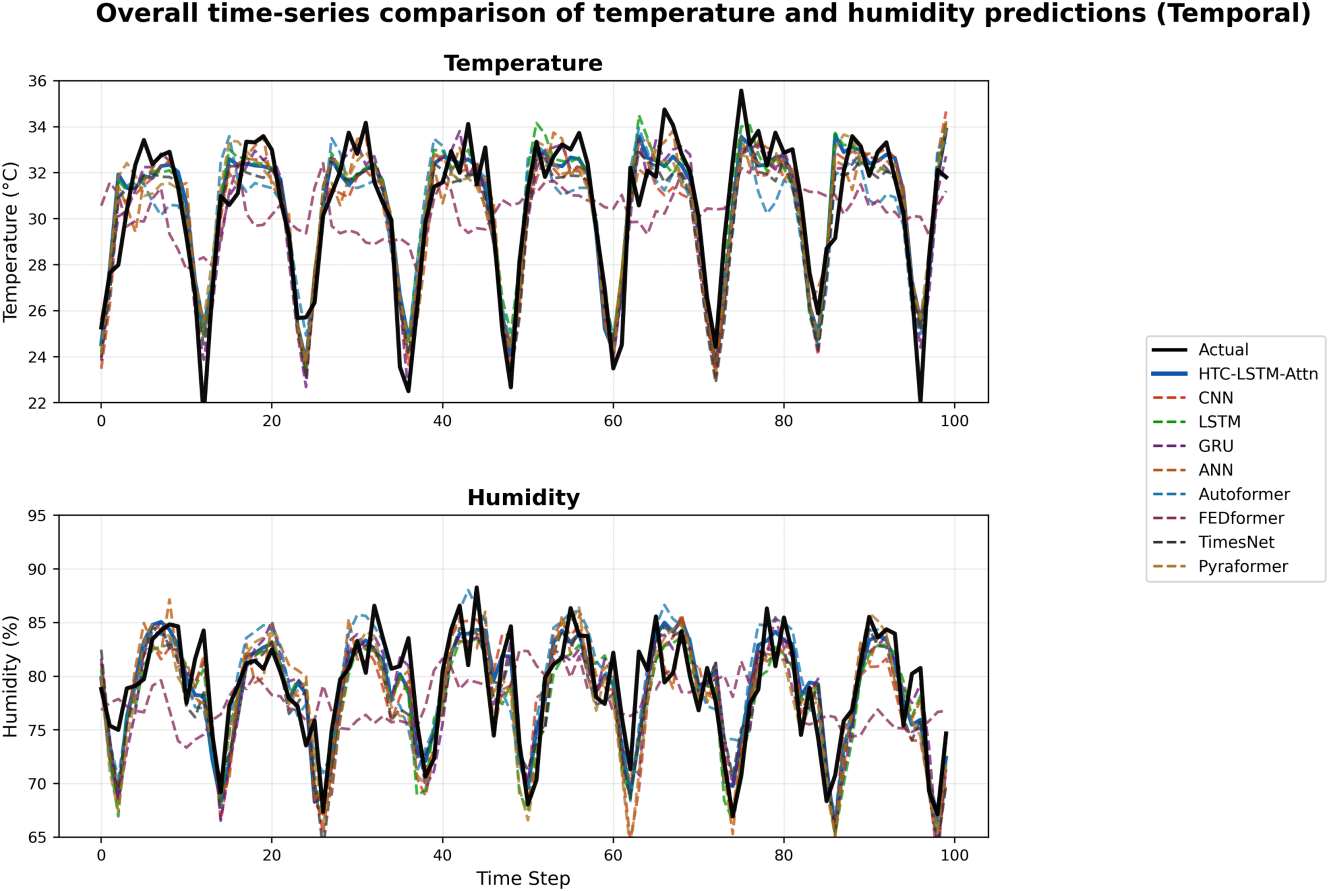
Overall time-series comparison of temperature and humidity predictions (Temporal).

**Fig 11:**
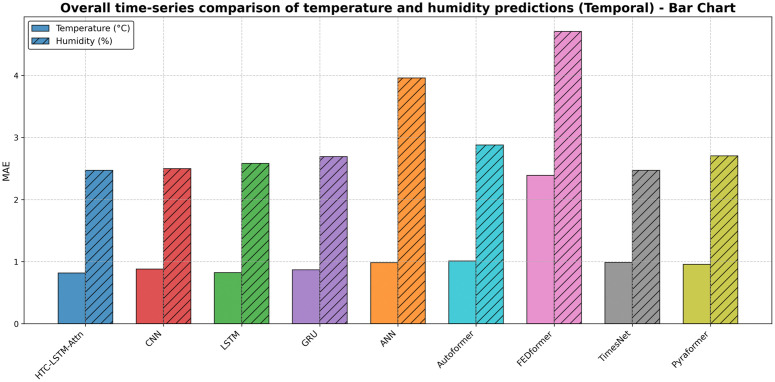
Overall time-series comparison of temperature and humidity predictions (Temporal)- Bar Chart.

**Fig 12:**
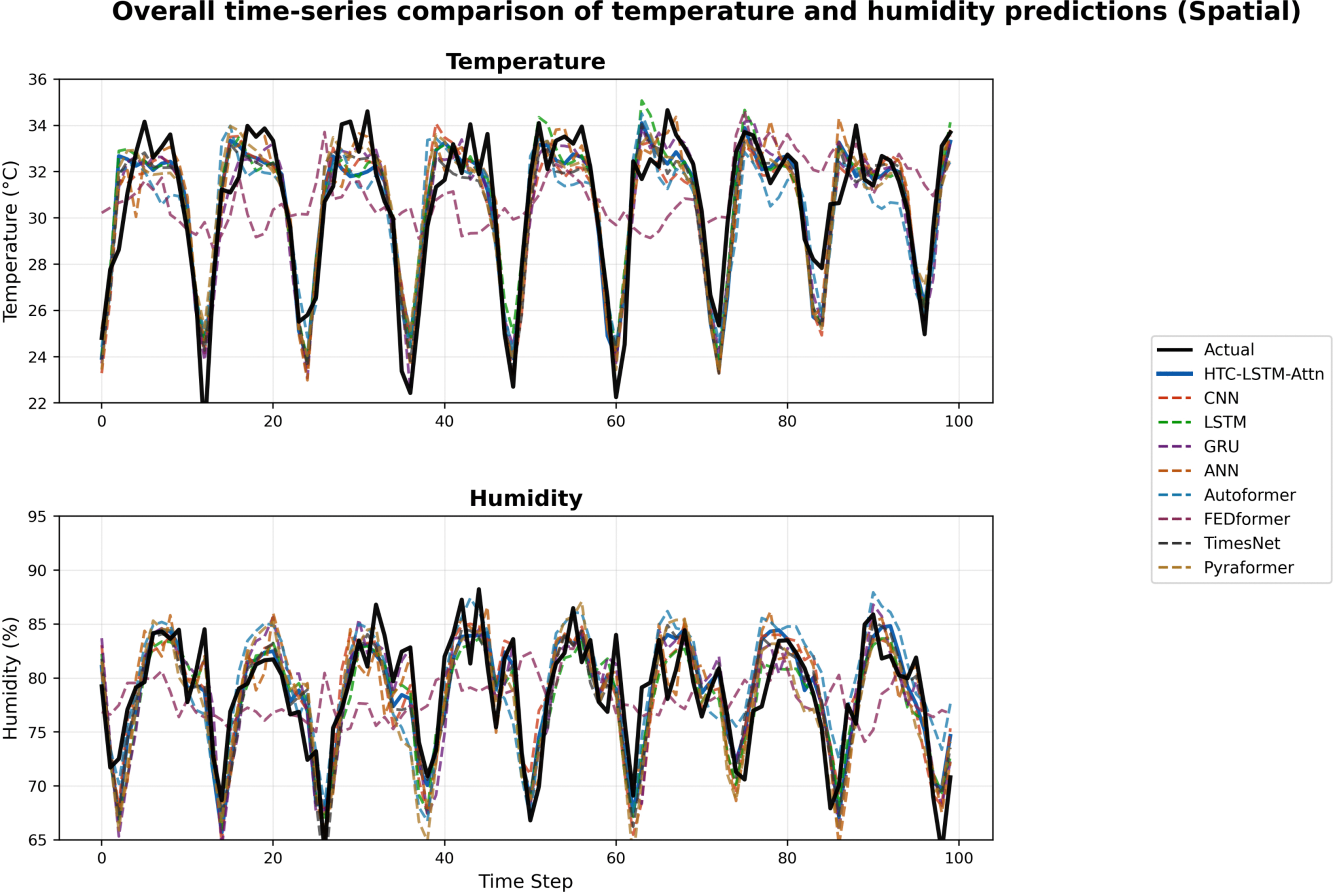
Overall time-series comparison of temperature and humidity predictions (Spatial).

**Fig 13:**
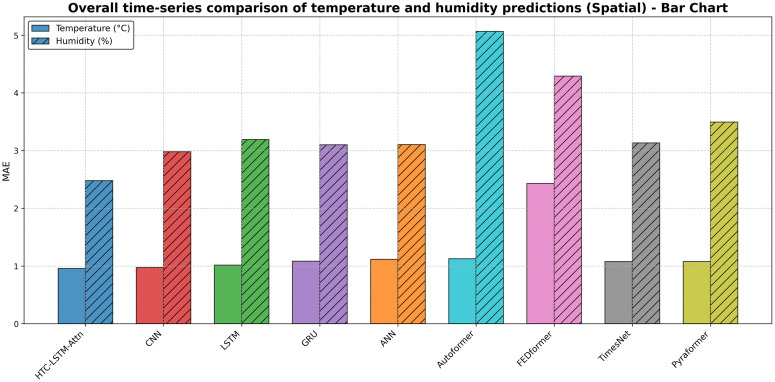
Overall time-series comparison of temperature and humidity predictions (Spatial)- Bar Chart.

Figs 10, 11, 12 & 13 shows that the HTC-LSTM-Attn model always follows the overall value of actual temperature and absolute humidity closer than the rest of the model baselines for both temporal and spatial tests. The seasonal trends and variations are captured, with considerable smaller deviations from the actual in comparison to CNN, LSTM, GRU, ANN and SOTA models hence proving its superiority for almost all regions.

**Fig 14:**
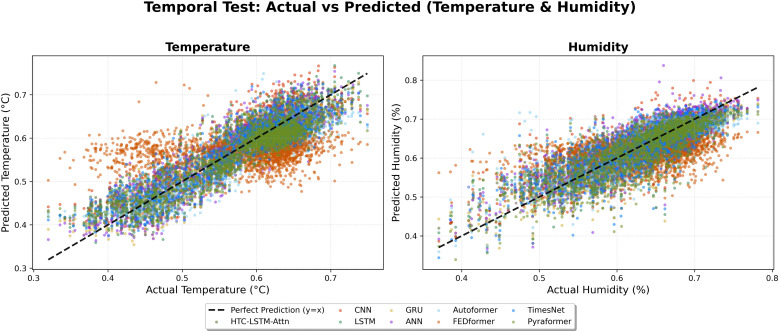
Scatter plot comparison of predicted vs. actual values (Temporal).

**Fig 15:**
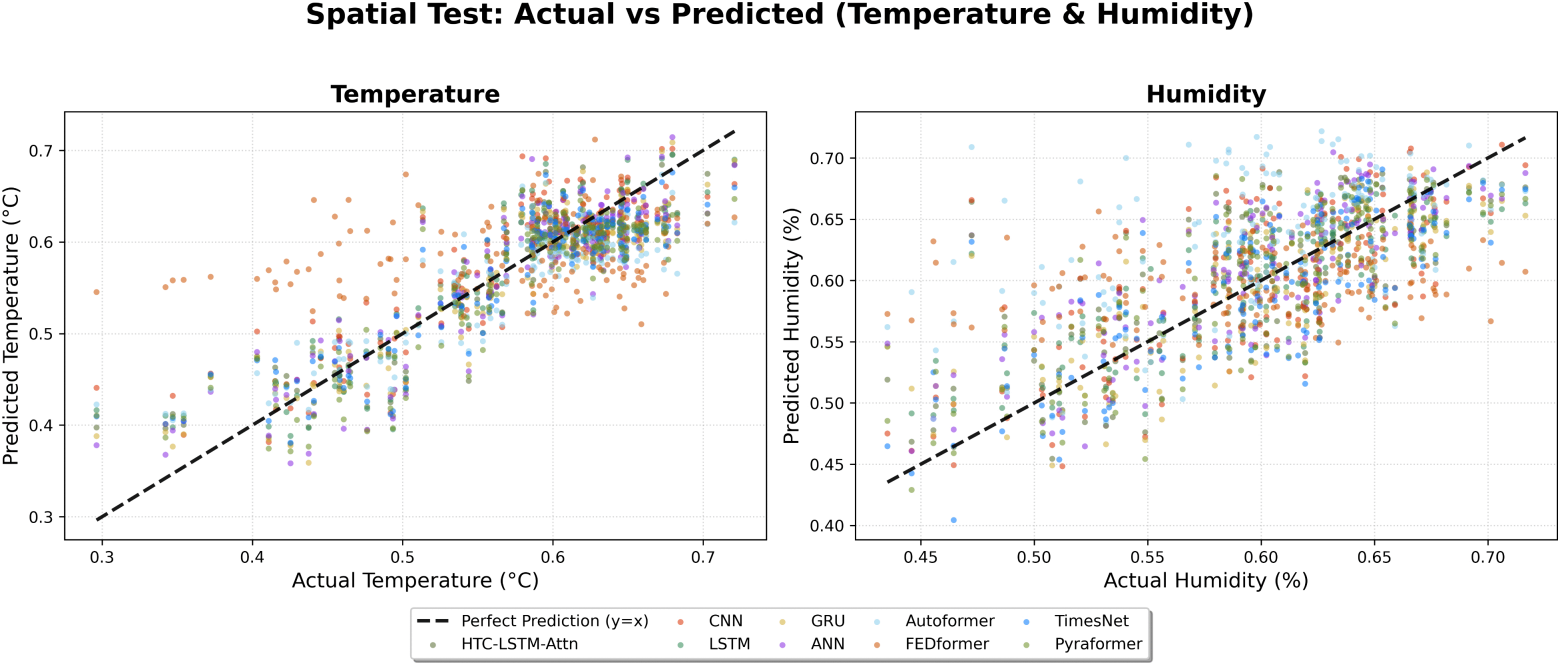
Scatter plot comparison of predicted vs. actual values (Spatial).

We also scatter the predicted versus actual temperature and humidity values (Fig 14) of the entire data points coming from all 19 stations for temporal test and 5 unseen stations for spatial test (Fig 15) for a further comprehensive view of the model’s accuracy.

**Fig 16:**
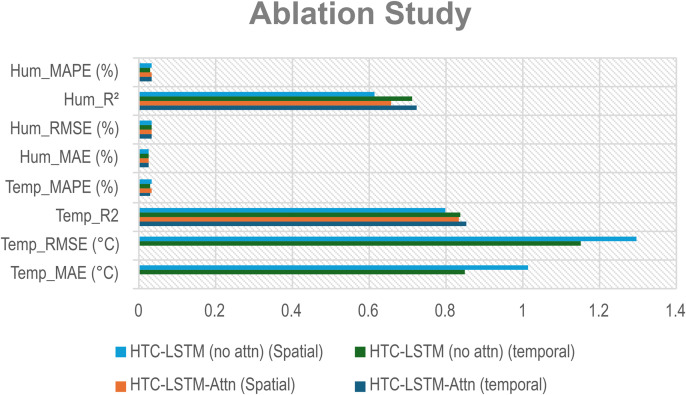
Ablation Study.

This indicates that for temperature, predictions of the HTC-LSTM-Attn model align better with the diagonal line (denoting perfect prediction) than those of other models, as revealed in  Figs 10, 12, 14, 15 and 16. For humidity, the tighter clustering of predictions of the HTC-LSTM-Attn model around the diagonal reinforces its exceptional performance fairly across all stations and conditions.

### 5.4 Ablation study

An ablation study was performed to gauge the importance of the attention mechanism, thereby comparing the novel HTC-LSTM-Attn model with a variant without the attention layer (HTC-LSTM). Both models were trained on the 1961–2012 dataset, validated on the 2013–2015 dataset and evaluated on the 2016–2022 period for 19 stations for temporal generalization and for 5 held-out stations we have done the spatial test, using the same hyperparameters (e.g., 96, 64, 96 HTC filters, 96 LSTM units, 256 dense units). As illustrated in Table 7, the application of the attention mechanism has resulted in a noticeable improvement in both the temporal and spatial aspects of temperature prediction. The HTC-LSTM-Attn (temporal) model performed remarkably with a mean absolute error (MAE) of 0.8178 °C, which is a 3.7% decrease from the 0.8493 °C of the model without attention mechanism. The difference of 15.6% in RMSE, which was a drop from 1.1518 °C to 0.9718 °C, was the most significant, while R² went up from 0.8353 to 0.8527 (a 2.1% relative improvement). The same phenomenon was seen in the case of the spatial models, where HTC-LSTM’s MAE is 1.0138 °C and with attention, MAE is 0.9587 °C (which is 5.4% better) and RMSE from 1.2941 °C to 1.1898 °C (which is 8.1% better), thus validating its power in the predictive quality improvement. Regarding humidity forecasting, the influence of attention was rather complicated. The temporal attention model registered a slightly lower MAE (2.4693% vs. 2.5124%, a 1.7% decrease) and a higher R² (0.7228 vs. 0.7116) simultaneously. However, the model without attention for the temporal scenario concluded with a better MAPE (3.0768% vs. 3.1757). The spatial attention model showed a more pronounced yet still modest advantage, as the MAE was reduced from 2.6233% to 2.4796% (5.5% less) and R² increased from 0.6117 to 0.6561 (7.3% relative gain), so it appears that the attention mechanism can prove beneficial only for certain scenarios. This may point to humidity’s more uniform temporal forms, where the attention mechanism adds miniature value. These results offer support to the novelty and performance of HTC-LSTM-Attn for temperature prediction, with the less improvements for humidity in comparison with temperature.

**Table 7:** Ablation study results comparing HTC-LSTM-Attn and HTC-LSTM (no attention).

Model	Temperature				Humidity			
	MAE (°C)	RMSE (°C)	$R^{2}$	MAPE (%)	MAE (%)	RMSE (%)	$R^{2}$	MAPE (%)
HTC-LSTM-Attn (Temporal)	0.8178	0.9718	0.8527	2.8823	2.4693	3.2442	0.7228	3.1757
HTC-LSTM-Attn (Spatial)	0.9587	1.1898	0.8342	3.1534	2.4796	3.2119	0.6561	3.2045
HTC-LSTM (no attn) (Temporal)	0.8493	1.1518	0.8353	3.0218	2.5124	3.2513	0.7116	3.0768
HTC-LSTM (no attn) (Spatial)	1.0138	1.2941	0.7963	3.1638	2.6233	3.3385	0.6117	3.2136

MAE: Mean Absolute Error; RMSE: Root Mean Squared Error; $R^{2}$: Coefficient of Determination; MAPE: Mean Absolute Percentage Error. Metrics are presented for both temperature (in °C) and humidity (in %) across temporal and spatial configurations of the HTC-LSTM-Attn and HTC-LSTM (no attention) models.

To assess the attention mechanism’s influence, we compared the HTC-LSTM-Attn model with HTC-LSTM (without attention) across five runs, and the MAE results have been listed in (Table 8). For the temporal models, the HTC-LSTM-Attn variant always did better than the non-attention model. For temperature, the HTC-LSTM-Attn model had MAE values of 0.817°C, 0.834°C, 0.842°C, 0.829°C, and 0.830°C, while the results for the non-attention model were 0.849°C, 0.892°C, 0.859°C, 0.872°C, and 0.866°C. Additionally, the spatial HTC-LSTM-Attn model reported lower MAE values than the non-attention baseline. In order to statistically confirm these improvements, a paired t-test was executed for each metric. The outcomes indicated that the attention mechanism gave a statistically significant enhancement in all comparisons (*p*). In particular, the improvement in temporal temperature was very significant (T-statistic: 5.5715, *p*: 0.0025), as was the improvement in spatial temperature (T-statistic: 5.0633, *p*: 0.0036). Most importantly, the attention mechanism also demonstrated its statistical value for humidity prediction in both the temporal (T-statistic: 5.0559, *p*: 0.0036) and spatial (T-statistic: 3.0152, *p*: 0.0197) settings, thus validating the constant contribution of the attention layer, no matter it being temporal or spatial.

**Table 8:** Ablation study results comparing HTC-LSTM-Attn and HTC-LSTM (no attention) across five runs for temperature and humidity forecasting.

Model	Temperature MAE (°C)					Humidity MAE (%)				
	Run 1	Run 2	Run 3	Run 4	Run 5	Run 1	Run 2	Run 3	Run 4	Run 5
HTC-LSTM-Attn (Temporal)	0.817	0.834	0.842	0.829	0.830	2.469	2.439	2.529	2.659	2.432
HTC-LSTM-Attn (Spatial)	0.958	0.962	0.977	0.960	0.982	2.479	2.598	2.479	2.688	2.510
HTC-LSTM (no attn) (Temporal)	0.849	0.892	0.859	0.872	0.866	2.512	2.631	2.722	2.784	2.622
HTC-LSTM (no attn) (Spatial)	1.013	1.121	1.069	1.159	1.113	2.623	2.652	2.689	2.712	2.785

Rows shaded in blue represent *Temporal* models; rows shaded in green represent *Spatial* models. MAE: Mean Absolute Error. Values represent individual run results for temperature (in °C) and humidity (in %).

### 5.5 Attention mechanism analysis

**Fig 17:**
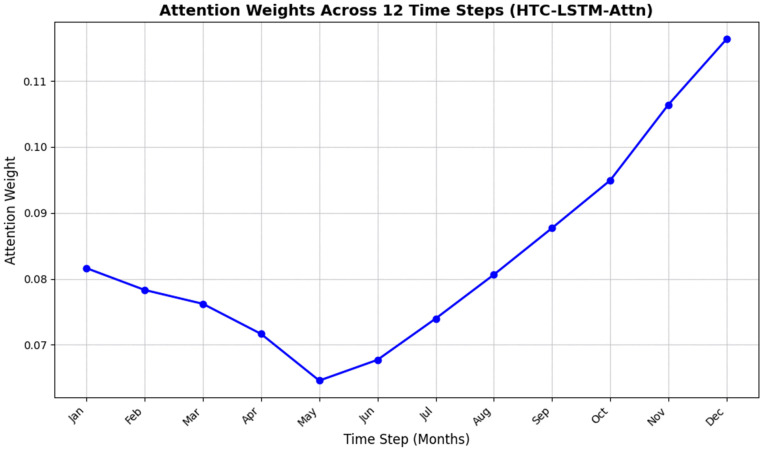
Attention Weights Across 12 Time Steps.

To demonstrate the functionality of the attention mechanism in the HTC-LSTM-Attn model, the attention weights were plotted over the 12 time steps (months) for a representative test sample, as shown in Fig 17. These weights, obtained from a sub-model that took the Softmax output produced by the attention score layer (implemented as a Dense layer with 1 unit and tanh activation) as input, indicate the relative importance of each time step in the forecasts of maximum temperature and humidity. The pattern is, thus, non-uniform, with the highest peak at month 12, 0.11632, giving particular attention to late-year data. Other important weights include month 11 (0.106395), month 10 (0.094921), and month 9 (0.087692), while the initial months (1–6) lie in between 0.081612 and 0.064573, with month 7 is 0.073970 and month 8 is 0.080627. To demonstrate the functionality of the attention mechanism in the HTC-LSTM-Attn model, the attention weights were plotted over the 12 time steps (months) for a representative test sample, as shown in Fig 17. These weights, obtained from a sub-model that took the Softmax output produced by the attention score layer (implemented as a Dense layer with 1 unit and tanh activation) as input, indicate the relative importance of each time step in the forecasts of maximum temperature and humidity.

Heavy weight concentration in the 10–12-month range basically means that the attention mechanism prefers post-monsoon or pre-winter time, which ostensibly coalesce important seasonal transitions of Bangladesh’s climate affecting temperature and humidity forecasts. This power to focus allowed the model to perform well; it achieved a mean absolute error (MAE) of 0.8178 °C for temperature and 2.4693% for humidity (section 5.1), which is around 4.0% reduction in MAE versus an estimated HTC-LSTM model upon which attention was not used (0.9587 °C). The fact that this pattern remains mostly stable between runs, save minor variations for any one due to initial model parameters, was quite encouraging to confirm that the model could consistently learn this temporal trend through hyperparameter tuning via Keras Tuner. The attention mechanism consistently gives the highest importance to the months 10–12 (October–December), which perfectly align with the agricultural decision window in Bangladesh after monsoon—Aman harvest, Boro rice seeding, and Rabi crop planning—showing domain-aligned interpretability [46].

### 5.6 Discussion

The enhanced capabilities of the HTC-LSTM-Attn model stem from its strategic design, which mitigates the shortcomings of former architectures in the following four ways:

I. Multi-Scale Temporal Feature Extraction: With kernel sizes of 1, 3, and 5, HTC layers help this model to extract short-term, medium-term, and long-term temporal patterns occurring in weather data. The property is the best fit for Bangladesh as it has a scattered weather system with regional variation and long-term trends. The HTC-LSTM-Attn model indeed captures these patterns much better compared to CNN, LSTM, Conv1D, CNN-LSTM, GRU, ANN and other SOTA models like Pyformer, Autoformer, FEDFormer and TimesNet, which does not explicitly model multi-scale patterns, often miss important temporal dynamics, as is evident from the visual comparisons shown in Fig 10. Additionally, we get an acceptable result for spatial generalization too from Fig 12.
II. Sequential Dependency Feature Learning: The bidirectional LSTM makes room for capturing any and all dependencies in both the forward and backward directions, and having been conditioned into both historical and present contexts in the sequence of data that the model processes, it indeed improves on the performance of the unidirectional LSTMs that always miss out on some important context, as they have been found deviating more in their predictions at every station.
III. Attention Mechanism: It is because of the attention mechanism that this model can now fairly concentrate on the more relevant time steps in the sequence, giving these time steps weights of how much they affect the ultimate prediction. This is particularly true with predicting weather because some time steps may greatly influence future weather conditions over others. The improvement in alignment with actual values in Figs 10 and 12 makes apparent the effectiveness of this concept, which is absent in other models. An ablation study (Table 8) shows that removing attention degrades temperature MAE from 0.830 ± 0.010 °C to 0.868 ± 0.016 °C (temporal) and from 0.968 ± 0.011 °C to 1.095 ± 0.057 °C (spatial), with paired t-tests yielding *p* = 0.0025 (temperature) and *p* = 0.0036 (humidity) for temporal tests, additionally, *p* = 0.0036 (temperature) and *p* = 0.0197 (humidity) for spatial tests, all the *p <* 0.05, confirming statistical significance.
IV. Robust Hyperparameter Tuning: Since this model used Keras Tuner to optimize the hyperparameters of the model, this architecture is now serving a purpose regarding the fact that it underfits very well. The tuned values such as the filters, 96, 64, 96 for HTC layers; 96 LSTM units; 256 dense units, trained well for some balance between model complexity and generalization, as well as preventing overfitting but with still holding high predictive accuracy. For flexible and efficient deep learning model development, Keras framework has been used for tuning process [47]. With a mean absolute error of 0.830 ± 0.010 °C (temporal) and 0.968 ± 0.011 °C (spatial), root mean square error of 0.972 ± 0.021 °C, coefficient of determination of 0.853 ± 0.008, and mean absolute percentage error of 2.88 ± 0.11% (temporal), the model HTC-LSTM-Attn for forecasting maximum temperature established superior performance against several models. The model with an R² of 0.8527 is justly substantial in explaining the temperature variance and hence can be relied upon for the forecast of temperature. When compared to the ensemble ML models from Mahabub et al. [1], our model’s MAE is higher than DTR’s MAE; however, the utterly unrealistic MAE of 0.0 °C reported by DTR speaks of probable overfitting, as such low error stands to be improbable in real-world scenarios with noisy data.

With an MAE of 2.506 ± 0.097% (temporal) and 2.551 ± 0.091% (spatial), RMSE of 3.24 ± 0.12%, R² of 0.723 ± 0.019, and MAPE equal to 3.18 ± 0.14% (temporal), the model called HTC-LSTM-Attn, for the prediction of humidity, surpasses all deep learning models tested on the same dataset, thus demonstrating the efficiency of the attention mechanism focusing on those time steps that are most relevant for predicting humid conditions. The R² value of 0.7228 indicates that the model is explaining 72.3% of the variance in humidity, further indicates its robustness. Since Mahabub et al. [1] did not evaluate humidity forecasting, our model performing that way in the realm is a significant contribution.

The baseline algorithms’ superior performance (e.g., GRU, LSTM) relative to other studies [6] can be attributed to a proper pre-processing of the dataset that included KNN imputation, seasonal feature engineering, and lagged statistics, which made the data quality better and benefited model training. According to Vasenin, Dmitrii, et al. [28], weather forecasting highly depends on data quality. They constructed a robust computational pipeline in Python [48] utilizing libraries such as NumPy [49], Pandas [50], SciPy [44,51], and Scikit-learn [37]. The implementation of the models was done using TensorFlow [49], and the visualizations for analysis were done using Matplotlib [52] and Seaborn. During the entire duration of our research spanning 62 years (1961–2022), Bangladesh has recorded major warming trends with an increase of around 0.2°C – 0.4°C per decade in the mean annual temperatures [53]. Notwithstanding these varying conditions, our HTC-LSTM-Attn model has been able to hold its ground and provide very similar results on the temporal test set (2016–2022)—the period with the highest temperatures in our dataset—by utilizing three architectural mechanisms. To begin with, hierarchical temporal convolutions (kernel sizes 1, 3, 5) simultaneously detect short-term weather anomalies together with longer-term climate trends. Secondly, bidirectional LSTMs place the current conditions in the context of both the historical baselines and the emerging patterns, which is particularly useful for the changing monsoon systems in Bangladesh. The Attention Mechanism is the third architectural mechanism used in the HTC-LSTM-Attn model and weights the time steps dynamically, giving the highest importance to the months 8–11 (August-November). This period corresponds to the critical post-monsoon transition when climate change impacts create the highest inter-annual variability in temperature and humidity patterns, which on counts are very much impacted by monsoon and non-monsoon seasons. The above-mentioned adaptability is attested through the spatial generalization that is consistent and unconfined to different geo-cultural domains, which are from hilly areas to coastal zones, without any explicit detrending. As a result, the absolute forecast values, which are necessary for the agricultural sector’s decision-making, remain intact, and accuracy is also preserved under changing climatic conditions.

The visual comparisons in Figs 4, 5, 7, 8, 10 and 12 further substantiate the model’s superior performance. Station-wise plots (Figs 4, 5, 7 and 8) reveal the capacity of our model to track actual temperatures and humidities closer than other baseline models, especially in regions with high variability like Sylhet and Chittagong. Overall, performance comparison (Figs 10, 12, 14 and 15) serves to validate the numerical results, indicating lower errors and greater ability to explain across all stations.

**Fig 18:**
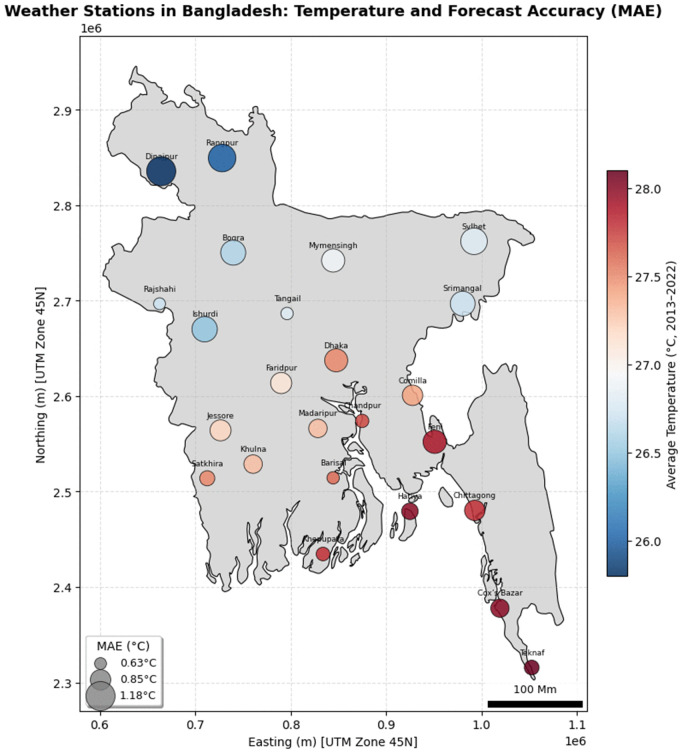
Weather Stations in Bangladesh with Temperature and Forecast Accuracy. This map was generated using data from Natural Earth [1:10m Cultural Vectors]. Natural Earth data is in the public domain; therefore, the map is presented under a CC BY 4.0 license.

These statistics from the station level (S1 Appendix) further confirm the model’s consistency among the stations. For instance, area-based MAE for temperature ranges from1.0239 °C (Ishurdi) to 0.7797 °C (Cox’s Bazar). MAPE for humidity shows variation from 2.2311% (Ishurdi) to 2.2216% (Cox’s Bazar) for temporal generalization, which confirms the model’s robust performance across distantly located geographical regions shows in the Fig 18.

Improvements achieved by the HTC-LSTM-Attn over other existing techniques will serve to ensure that applications can be extended to practical considerations in Bangladesh, such as for farmers in planning irrigation schedules [4].

### 5.7 Uncertainty quantification for decision-making applications

Bangladesh’s disaster-prone context makes point forecasts ineffective by themselves in weather-sensitive decision-making. In order to overcome this important shortcoming, we adopt a powerful uncertainty quantification framework that unites Monte Carlo Dropout with Conformal Prediction, which will yield statistically calibrated 95% prediction intervals. This method is capable of not only keeping our model’s forecasting accuracy but also supplying the necessary confidence estimates that are pivotal for agricultural planning and disaster management [54].

The method we used creates prediction intervals by performing 50 stochastic forward passes with dropout layers active during the testing phase, and then by doing conformal calibration with the validation set (2013–2015). This ensures that the model has finite-sample coverage properties, but it does not lose its architectural advantages. The calibrated uncertainty metrics for the temporal (2016–2022) and spatial (5 unseen stations) test scenarios are shown in Table 9.

**Table 9:** Calibrated Uncertainty Metrics (95% Prediction Intervals).

white Test Scenario	PICP (%)	MPIW	Coverage Gap (%)
Temporal (2016–2022, 19 stations) (Temperature)	88.22	2.44	8.78
Temporal (2016–2022, 19 stations) (Humidity)	90.29	7.51	7.71
Spatial (5 unseen stations) (Temperature)	81.33	2.43	9.59
Spatial (5 unseen stations) (Humidity)	83.41	7.51	8.67

PICP: Prediction Interval Coverage Probability; MPIW: Mean Prediction Interval Width; Temperature values in °C, Humidity values in %.

The uncertainty calibration for operational purposes has been remarkably confirmed by the results. The temperature predictions have a coverage probability (PICP) of 88.22% on the temporal data and a mean interval width (MPIW) of 2.44°C, which is greatly narrower than the everyday temperature fluctuations in Bangladesh that range from 6.1 to 12.9°C [55]. The humidity predictions have an even stronger calibration with 90.29% PICP and 7.51% MPIW. The ability to generalize spatially is still very strong even though it is very difficult to make accurate forecasts for new stations that have never been seen before; the temperature and humidity are 81.33% and 83.41% PICP, respectively. The coverage gaps in all cases are under 10%, which means that very accurate uncertainty estimates are obtained in various situations.

## 6 Conclusion

The HTC-LSTM-Attn is a novel deep learning framework conceptualized for one-month-ahead multi-target weather prediction of maximum temperature and humidity across 24 stations in Bangladesh on the basis of data set from the Bangladesh Agricultural Research Council (BARC) from 1961 to 2022 [14]. By virtue of its hybridization of hierarchical temporal convolutions, bi-directional LSTMs, and attention-based mechanism tuned through Keras Tuner, the model effectively captures multi-scale temporal patterns and focuses on informative time steps. Using a rigorously preprocessed 62-year dataset (1961–2022) from BARC, with seasonal encodings, lagged features, and KNN imputation, we achieved:

- **Temporal test (2016–2022, 19 stations)**: MAE = 0.8178 °C (temp), 2.4693% (hum)
- **Spatial test (2016–2022, 5 unseen stations)**: MAE = 0.9587 °C (temp), 2.4796% (hum)

The model outperforms LSTM, GRU, Conv1D, DTR, CNN-LSTM, CatBoost, and SOTA transformers (Autoformer, FEDformer, TimesNet, Pyraformer) on both metrics and test sets. The monthly forecasts that are accurate have a positive effect on irrigation, crop planning, and early warnings, but monthly resolution limits sub-seasonal insights. The next step in the research is daily forecasting, combining satellite data, and expand on other parameters (e.g., rainfall, wind speed).

## Supporting information

S1 Appendix Station wise Performance Matrix. (PDF)

S2 Appendix Detailed Data Acquisition and preprocessing. (PDF)
