# Supplementary material for: Redefining multi-target weather forecasting with a novel deep learning model: Hierarchical temporal convolutional long short-term memory with attention (HTC-LSTM-Attn) in Bangladesh
Source: PLoS One. 2026 Mar 23;21(3):e0342431. doi: 10.1371/journal.pone.0342431 (PMC13008104; doi:10.1371/journal.pone.0342431)
Supplement: S1 Appendix — (PDF) [file pone.0342431.s001.pdf]

Appendix:

Table A1: Station wise Performance Matrix

Temporal Generalization for 19 Stations:

Station Code	Station	HTC_LSTM_Attn_ Temp_MAE	HTC_LSTM_Attn_ Temp_RMSE	HTC_LSTM_Attn_ Temp_R2	HTC_LSTM_Attn_ Temp_MAPE	HTC_LSTM_Attn_ Hum_MAE	HTC_LSTM_Attn_ Hum_RMSE	HTC_LSTM_Attn_ Hum_R2	HTC_LSTM_Attn_ Hum_MAPE
10208	Rangpur	1.111470877	1.412562784	0.8137934523	3.816168726	2.69703615	3.307055021	0.4781429211	3.393837217
10408	Bogra	1.008387618	1.343460632	0.8170055476	3.429042873	3.21251354	4.103470342	0.5872193828	4.218680854
10609	Mymensingh	0.939044359	1.193767959	0.8328658862	3.225033283	2.022995461	2.659900855	0.5898935569	2.486140611
10705	Sylhet	1.071336351	1.427775408	0.6782161544	3.549891919	3.083489384	3.671480037	0.7424749866	3.971663091
10910	Ishurdi	1.023915265	1.23217745	0.8915011051	3.393004354	2.231170273	2.734482024	0.7478881641	2.896345843
11111	Dhaka	0.9334865401	1.162363784	0.8541124864	3.078244803	3.394776488	4.067687606	0.7464639185	4.785082858
11313	Comilla	0.8469081222	1.053371722	0.8432847317	2.800294577	2.167366808	2.850873899	0.6395036211	2.774262422
11407	Jessore	0.8648270067	1.020193912	0.9075098671	2.775984124	2.043877958	2.628907101	0.7232409374	2.614530555
11505	Faridpur	0.8678028997	1.04964359	0.890790317	2.848457251	2.216014616	2.925560207	0.7357942214	2.926149984
11604	Khulna	0.7859714567	0.939831166	0.917209076	2.578888467	2.086184786	2.615224245	0.7588975551	2.703683124
11610	Satkhira	0.698061214	0.8415303964	0.9333513472	2.285631299	1.936802555	2.335586071	0.7623296232	2.512853966
11704	Barishal	0.7220843612	0.8596723803	0.8987408015	2.309516148	2.00624541	2.554496702	0.7253141863	2.449977484
11706	Bhola	0.8355309804	1.023203617	0.8541545455	2.699571592	2.066627998	2.739956895	0.6515186923	2.566389006
11809	M. Court	0.832740736	1.012294376	0.874657361	2.734201359	2.641046855	3.368709264	0.5806025385	3.337512423
11814	Hatiya	0.7353494835	0.9326966239	0.8470110149	2.436558493	2.117479468	2.596987598	0.6689570537	2.493552638
11912	Sitakunda	0.9680246936	1.166942822	0.6960466963	3.103464275	3.576776115	4.947155618	0.5170674138	4.920230696
11916	Sandwip	0.7815359709	0.973013388	0.8043537068	2.533423707	2.042408795	2.602576689	0.6799519878	2.515541392
11921	Chittagong	0.8456524552	1.015816217	0.8056896742	2.764601031	2.512362624	3.200810805	0.7636111463	3.313591407
11927	Cox's Bazar	0.7797596825	0.9386499433	0.752676529	2.517629537	2.221645571	2.761082865	0.8445290201	2.923629564

Spatial Generalization for 5 held-out Stations:

Station Code	Station	HTC_LSTM_Attn_Temp_MAE	HTC_LSTM_Attn_Temp_RMSE	HTC_LSTM_Attn_Temp_R2	HTC_LSTM_Attn_Temp_MAPE	HTC_LSTM_Attn_Hum_MAE	HTC_LSTM_Attn_Hum_RMSE	HTC_LSTM_Attn_Hum_R2	HTC_LSTM_Attn_Hum_MAPE
10120	Dinajpur	1.181234359	1.503067345	0.8155824353	4.078933566	2.446200244	3.008191158	0.640340531	3.105241649
10724	Srimangal	1.000708062	1.19717833	0.820951408	3.26038601	2.3012758	2.831422932	0.6353527645	2.827774572
11513	Madaripur	0.7845270199	0.9332648876	0.9115502421	2.553915276	1.639962959	2.225252534	0.7763504175	2.065808976
11805	Feni	0.9437520112	1.155738905	0.7923330265	3.052355159	2.49237704	3.123250219	0.6724410449	3.274545492
12007	Rangamati	0.96071773	1.177381578	0.7997132825	3.083968664	3.730058462	4.676497266	0.2956968944	5.078236155
