# Supplementary material for: Redefining multi-target weather forecasting with a novel deep learning model: Hierarchical temporal convolutional long short-term memory with attention (HTC-LSTM-Attn) in Bangladesh
Source: PLoS One. 2026 Mar 23;21(3):e0342431. doi: 10.1371/journal.pone.0342431 (PMC13008104; doi:10.1371/journal.pone.0342431)
Supplement: S2 Appendix — (PDF) [file pone.0342431.s002.pdf]

S2 Appendix: Detailed Data Acquisition and Preprocessing Pipeline

A. Data Acquisition and Consolidation

The Bangladesh Agricultural Research Council (BARC) provided monthly weather records from January 1961 to December 2022, covering 35 stations across Bangladesh. Data were supplied as separate CSV files: *Solar_Radiation_Report.csv*, *PET_Report.csv*, *Sunshine_Report.csv*, *WindSpeed_Report.csv*, *Cloud_Cover_Report.csv*, *Humidity_Report.csv*, *Rainfall_Report.csv*, and *Maximum_Temperature_Report.csv*.

All computations were performed in Google Colab with python 3.12, T4 GPU With 51 GB RAM.

Each file was merged using **Station Code**, **Year** (1961–2022), and **Month** (1–12) as the primary keys. Preprocessing steps included data consolidation, cleaning, and enhancement to provide a single data set. The merged dataset was stored as *Organized_Weather_Data.csv* and used in all forecasting experiments.

B. Feature Engineering

To incorporate seasonality and temporal structure, the following features were generated:

1. Seasonal encodings

$$\text{Month}_{\text{sin}} = \sin\left(2\pi\frac{m}{12}\right), \quad \text{Month}_{\text{cos}} = \cos\left(2\pi\frac{m}{12}\right)$$

2. Lagged features For target variables (temperature, humidity), 1-, 2-, and 3-month lags:

$$x_{t-\ell}, \quad \ell \in \{1, 2, 3\}$$

3. Rolling statistics month moving averages and standard deviations(i.e, 3 month):

$$\text{roll\_mean}_3 = \frac{1}{3}(x_{t-2} + x_{t-1} + x_t)$$

C. Missing Data Imputation

Missing values were filled using K-Nearest Neighbors (KNN, $k = 5$):

$$\hat{x}_i = \frac{1}{k} \sum_{j \in \mathcal{N}_k(i)} x_j$$

D. Outlier Detection

Outliers were identified using the IQR method:

$$x < Q_1 - 1.5 \times IQR \quad \text{or} \quad x > Q_3 + 1.5 \times IQR$$

Detected outliers were replaced with the nearest valid value.

E. Data Quality Check

A time series was plotted for the stations with Matplotlib and Seaborn for visual validation of the imputed values .

F. Implementation Details

The data preprocessing was performed in Python using Pandas, NumPy, SciPy, and Scikit-learn for data handling, feature engineering, statistical tests, KNN imputation, outlier detection, and scaling. The resulting Organized Weather Data is optimized for training the ability of the HTC-LSTM-Attn model to seize the hierarchical and temporal weather patterns in Bangladesh.
